# Global gene expression analysis using RNA-seq uncovered a new role for SR1/CAMTA3 transcription factor in salt stress

**DOI:** 10.1038/srep27021

**Published:** 2016-06-02

**Authors:** Kasavajhala V. S. K. Prasad, Amira A. E. Abdel-Hameed, Denghui Xing, Anireddy S. N. Reddy

**Affiliations:** 1 Department of Biology and Program in Molecular Plant Biology, Colorado State University, Fort Collins, CO, 80523, USA

## Abstract

Abiotic and biotic stresses cause significant yield losses in all crops. Acquisition of stress tolerance in plants requires rapid reprogramming of gene expression. SR1/CAMTA3, a member of signal responsive transcription factors (TFs), functions both as a positive and a negative regulator of biotic stress responses and as a positive regulator of cold stress-induced gene expression. Using high throughput RNA-seq, we identified ~3000 SR1-regulated genes. Promoters of about 60% of the differentially expressed genes have a known DNA binding site for SR1, suggesting that they are likely direct targets. Gene ontology analysis of SR1-regulated genes confirmed previously known functions of SR1 and uncovered a potential role for this TF in salt stress. Our results showed that *SR1* mutant is more tolerant to salt stress than the wild type and complemented line. Improved tolerance of *sr1* seedlings to salt is accompanied with the induction of salt-responsive genes. Furthermore, ChIP-PCR results showed that SR1 binds to promoters of several salt-responsive genes. These results suggest that SR1 acts as a negative regulator of salt tolerance by directly repressing the expression of salt-responsive genes. Overall, this study identified SR1-regulated genes globally and uncovered a previously uncharacterized role for SR1 in salt stress response.

Abiotic stresses, such as drought, cold, heat and salinity, and biotic stresses caused by pathogenic bacteria, viruses and fungi, limit plant growth and development resulting in significant yield losses in crop plants^1^,^2^,^3^. Acquisition of tolerance to these stresses and other adverse environmental conditions requires coordinated regulation of a multitude of biochemical and physiological changes, and a vast majority of these changes rely on stress-dependent reprogramming of gene expression^4^,^5^,^6^,^7^,^8^,^9^. The alterations in gene expression patterns are largely responsible for plants’ ability to cope with the adverse environmental factors. Previous studies have shown that Ca^2+^ is one of the key messengers in mediating stress responses^7^,^10^,^11^. Stress-induced changes in cellular Ca^2+^ are perceived by Ca^2+^ sensors such as calmodulin (CAM), which in turn regulate diverse processes including gene expression^7^. Signal responsive (SR) proteins, which are also referred to as CAMTAs (CAM-binding Transcriptional Activators), are a class of highly conserved Ca^2+^/CAM-binding transcription factors (TFs) in plants and animals^12^,^13^,^14^,^15^,^16^,^17^. In Arabidopsis there are six SR family TFs (SR1 to SR6) and expression of these genes is regulated by diverse biotic and abiotic stresses, as well as hormones^12^,^13^,^18^,^19^,^20^. All members of SR/CAMTA family TFs have a DNA binding domain called CG-1 at the N-terminus, which binds to *CGCG* or *CGTG* core motifs^21^,^22^,^23^,^24^, a TIG (an immunoglobulin–like fold) domain that is involved in non-specific DNA binding, several ankyrin repeats that are responsible for protein-protein interactions, followed by five tandem repeats of Ca^2+^-independent CAM binding domains (IQ motifs), and a Ca^2+^-dependent CAM binding domain^7^,^11^. SR1 (also known as CAMTA3) is one of the well-studied members of the SR family TFs. The core DNA binding motif of SR1 is part of a rapid stress response element (RSRE - *VCGCGB*) found in the promoters of many genes that are rapidly activated in response to stress^25^,^26^. It has been shown that SR1 can activate reporter genes driven by RSRE in a Ca^2+^-dependent manner^26^, further suggesting the role of SR1 in stress-induced gene expression through Ca^2+^. Recent genetic screens also confirmed that SR1 is an important component in RSRE-driven gene expression^27^.

Several studies with SR1 have shown that it functions as a negative regulator of plant immunity in Arabidopsis^28^,^29^,^30^, a positive regulator of insect resistance^31^,^32^ and cold-induced gene expression^24^,^33^. A rice CAMTA (*OsCBT)* also functions as a negative regulator of disease resistance against *Xanthomonas oryzae* and *Magnaporthe grisea*^34^. Although SR1 has been shown to play important regulatory roles in plant immunity, herbivory and cold-induced gene expression, the full set of SR1-regulated genes is largely unknown. A previous microarray study performed with wild type and *SR1* mutant reported only about 100 SR1-regualted genes^29^. However, in that study a complemented line was not included. Here we sequenced the transcriptomes of wild type, *SR1* mutant and a complemented line using RNA-seq and identified about 3000 SR1-regulated genes. By analyzing the promoters of all SR1-regulated genes for the presence of known SR1 binding sites, we identified potential direct targets of SR1. Comprehensive analysis of SR1-regulated genes confirmed its known roles and uncovered a previously uncharacterized role for SR1 in salt stress. Furthermore, our results established that SR1 is a negative regulator of salt stress.

## Results

### Loss of SR1 resulted in misregulation of about 3000 genes

**Figure 1:**
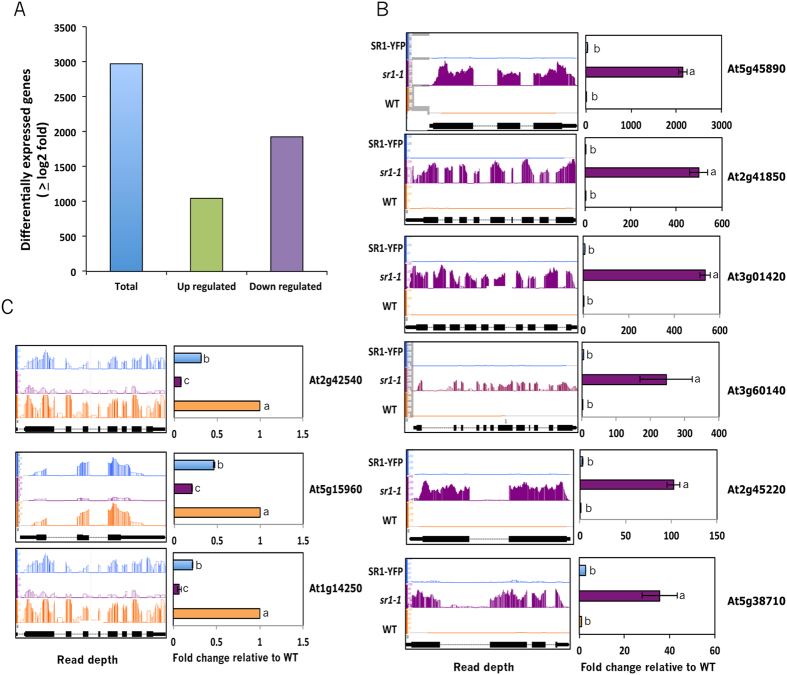
SR1-regulated genes in Arabidopsis. (**A**) Total DE, up- or down-regulated genes. (**B**) RT-qPCR validation of randomly selected up-regulated genes. (**C**) RT-qPCR of randomly selected down-regulated genes. Left panels in (**B,C**) show relative sequence read abundance (Integrated Genome Browser view) as histograms in WT, *sr1-1* and SR1-YFP lines. The Y-axis indicates read depth with the same scale for all three lines. The gene structure is shown below the read depth profile. The lines represent introns and the boxes represent exons. The thinner boxes represent 5′ and 3′ UTRs. Right panels in (**B,C**) show fold change in expression level relative to WT. WT values were considered as 1. Student t-test was performed and significant differences (P < 0.05) among samples are labeled with different letters. The error bars represent SD.

Although SR1 TF is known to regulate multiple stress responses in plants, an in-depth study of SR1-regualted genes (direct or indirect) in the genome using deep sequencing of transcriptomes has not been performed. Here we performed RNA-seq analysis of gene expression with RNA from wild type, *SR1* loss-of-function mutant and a complemented line in which the mutant phenotypes are rescued^28^,^31^. Prior to RNA-seq, genotypes of all three lines were verified by genomic PCR and RT-qPCR (Supplementary Fig. S1). In the complemented line, the expression of *SR1* at the protein level was also confirmed (Supplementary Fig. S1). For each line, two biological replicates were sequenced using Illumina platform. About 37 to 45 million high quality reads (FastQC quality score is >36) were obtained for each replicate (Supplementary Table S1). About 80 million reads for each line were used for gene expression analysis. Around 94% of reads from each sample were mapped to the Arabidopsis genome (TAIR10) (Supplementary Table S1). Of these, ~90 to 92% of the reads were uniquely mapped to a single location. The expression of each transcript in each sample was measured by the number of reads per kilobase per million reads (RPKM). A very high linear correlation was observed in the expression of genes among the replicates indicating that there are no significant differences in gene expression among the biological replicates (Supplementary Fig. S2). The R^2^ values were between 0.87 and 0.9 for the replicates of all three lines (Supplementary Fig. S2). However, there was a substantial effect of *SR1* loss on gene expression as evident from linear regression values when compared to WT (Supplementary Fig. S2B). Furthermore, expression of *SR1* in *sr1* mutant background significantly restored gene expression changes observed in the mutant (Supplementary Fig. S2). Using the Cufflinks package we identified differentially expressed (DE) genes by comparing the transcriptomes of the mutant and wild type. A total of 2973 genes (Adj. P < 0.05 and fold change >2) were misregulated in *sr1* as compared to the WT (Additional File 1, Sheet 1). Expression of about ~85% of DE genes was partially or fully restored to wild type level (Supplementary Fig. S3 and Additional File 1, Sheet 2). These results suggest that the DE genes in the mutant are either direct or indirect targets of SR1 and that the loss of this TF has substantial effect on expression of large number of genes (Fig. 1A). Among the DE genes, 1046 were up-regulated whereas 1927 were down-regulated (Fig. 1A). Using RT-qPCR we validated the expression of 9 randomly selected DE genes. The RT-qPCR results corroborated RNA-seq data and the observed changes in the mutant were fully or partially restored in the complemented line (Fig. 1B,C). In addition, expression of several other DE genes involved in salt stress was also verified by RT-qPCR (see below).

### GO term enrichment of DE genes for biological processes

SR1 is known to function in plant immunity, herbivory and cold-regulated gene expression^24^,^28^,^29^,^31^,^33^. To verify if the DE genes function in these processes and to gain some insight into other functions of SR1, we performed Gene ontology (GO) enrichment analysis using the whole genome as background. Two methods, AgriGO and GeneCoDis, for singular GO term enrichment analysis yielded similar results with slight variation in the number of GO terms and the order of significance (data not shown). Results obtained with GeneCoDis are presented in Supplementary Fig. S4. A total of 81 GO terms for biological processes were enriched (Supplementary Fig. S4A and Additional File 2, Sheet 1). Consistent with the previous known functions of SR1, GO terms related to plant response to pathogens and abiotic factors were among the enriched terms. Analysis of the up- and down-regulated genes separately resulted in enrichment of 95 and 52 GO terms, respectively (Supplementary Fig. S4 and Sheets 2 and 3 in Additional File 2). Majority of the up-regulated GO terms are associated with plant defense response to biotic factors. In addition, GO terms “response to salt stress” and “response to water deprivation” are also highly enriched in the up-regulated genes. (Supplementary Fig. S4B and Additional File 2 Sheet 2). A significant enrichment of GO terms associated with abiotic factors such as “response to cold” and “response to water deprivation” was observed in down-regulated genes (Supplementary Fig. S4C and Additional File 2 Sheet3).

### DE genes are enriched for SR1 binding motif

**Figure 2:**
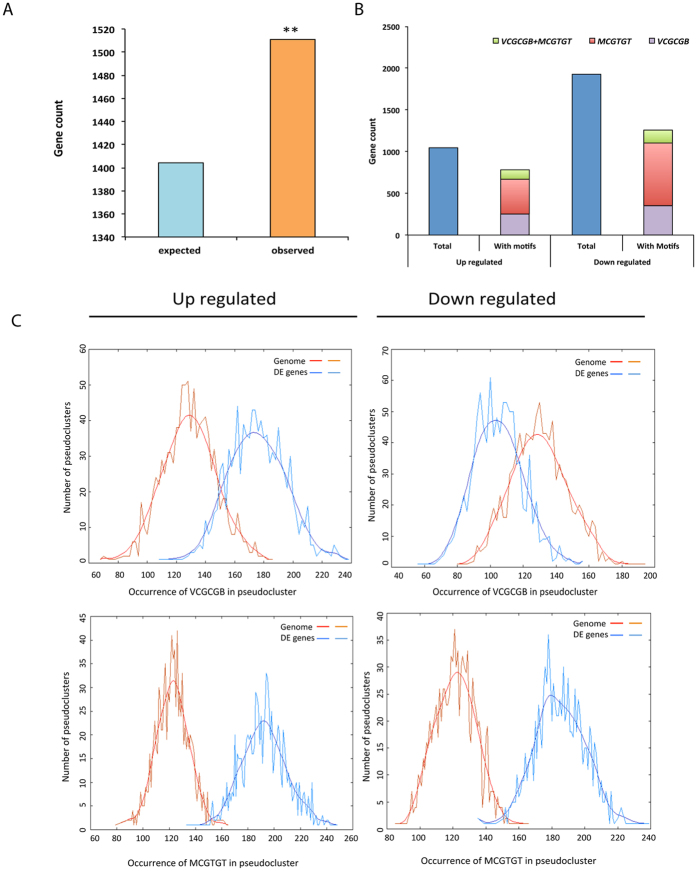
SR1-binding sites in the promoters of up- and down-regulated genes. (**A**) A significant enrichment of the SR1 binding motifs (*VCGCGB* + *MCGTGT*) in the upstream (−1000 bp) of TSS of all DE genes. Asterisks on the bar represent significant overrepresentation of binding sites with a P < 0.0001. (**B**) Total number of up- and down-regulated genes and the number of the SR1-regulated genes that contain SR1 binding sites *VCGCGB* or *MCGTGT* or *MCGCGT* + *VCGCGB* in the −1000 bp promoter region. (**C**) Top panel: POBO analysis of RSRE (*VCGCGB)* motif in the −500 bp upstream of TSS. 1000 pseudoclusters were generated from top 500 genes from up- or down-regulated genes and genome background. The jagged lines show the motif frequencies from which the best-fit curve is derived. RSRE element is significantly overrepresented with a two-tailed P < 0.0001 in the upstream sequences of up-regulated genes but not with down-regulated genes. Bottom panel: POBO analysis of a second SR1 recognition motif (*MCGTGT*) using the −500 bp upstream of TSS in 1000 pseudo clusters of top 500 DE genes and genome background. The jagged lines show the motif frequencies from which the best-fit curve is derived. SR1 binding sites are significantly over represented (two-tailed P < 0.0001).

Previous studies using an oligo selection method and electrophoretic mobility shift assays showed that SR1 binds to *VCGCGB* (where V = A, C or G; B = C, G or T) and *MCGTGT* (where M = A or C) motifs in the promoter regions of SR1-regulated genes^11^,^23^,^24^,^28^,^35^. The rapid activation of the general stress-responsive genes is also mediated through RSRE element (*VCGCGB)*, as promoters of many of these genes exhibit significant enrichment for this motif^25^,^26^. Here we determined whether the promoter regions of DE genes are enriched for the *VCGCGB* and *MCGTGT* motifs. As shown in Fig. 2A, both these motifs are enriched in the promoters (−1000 bp upstream of translation start site -TSS) of all DE genes (P < 0.0001). As significant enrichment for SR1 binding motifs was observed, we further checked for actual number of differentially up- or down-regulated genes that contained *VCGCGB* and/or *MCGTGT* in their promoters. Out of 1046 genes that are up-regulated, 665 (~64%) contained a minimum of one motif of either type (Fig. 2B, and Additional File 3). Of these, 37% contain *VCGCGB*, 39% have *MCGTGT* and 16% have both *VCGCGB* and *MCGTGT* (Fig. 2B). Similarly, out of 1927 down-regulated genes, 1098 (57%) have one or more of these motifs. Of these, 32% have *VCGCGB*, 67% have *MCGTGT* element and 13% have both (Fig. 2B, and Additional File 3). Together, these results indicate that a significant number (59%) of DE genes are likely direct targets for SR1. To identify if these motifs are enriched in the promoters of up- or down-regulated genes, we further analyzed the promoters using POBO analysis with upstream regions of top 500 up-regulated or down-regulated gens using the whole genome as background. This analysis revealed a significant enrichment (P < 0.0001) of both *cis*-elements (*VCGCGB* and *MCGTGT*) in the up-regulated genes whereas in the down-regulated genes only *MCGTGT* was enriched (Fig. 2C).

### GO term enrichment of SR1 binding motif-containing genes

**Figure 3:**
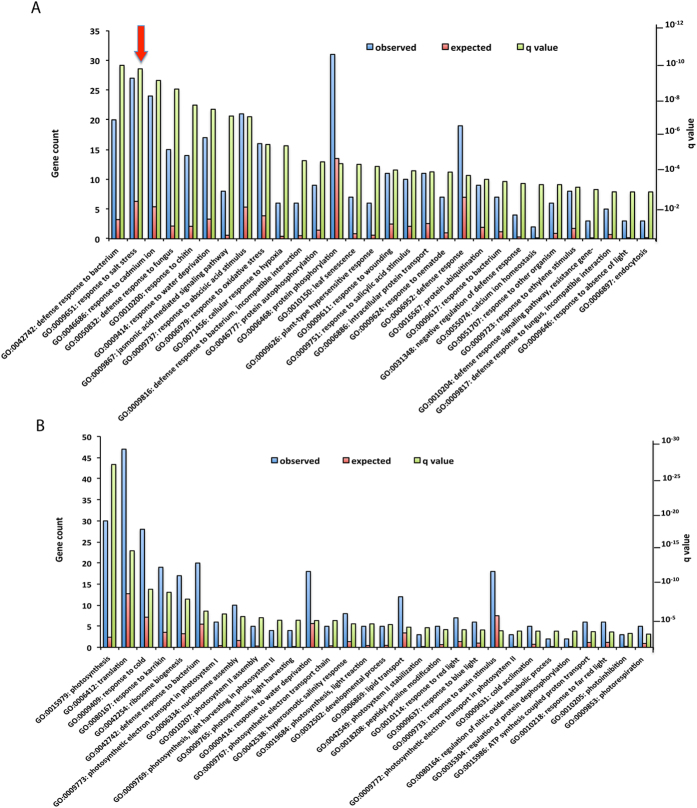
GO term enrichment analysis. GO term enrichment analysis for biological processes of (**A**) up- and (**B**) down-regulated genes. For each GO term, the expected and observed gene numbers along with the statistical significance (q-value) for the enrichment is presented. Observed: Number of DE genes associated with a GO term for biological processes. Expected: Number of genes expected for each GO term in the genome. “Response to salt stress” GO term is indicated with an arrow.

To understand the biological role of putative direct targets of SR1, we performed a separate GO enrichment analysis using either up- or down-regulated genes that have one or more SR1 binding motifs. In the up-regulated genes, 61 GO categories showed significant enrichment (Additional File 4). Top 30 GO categories are represented in Fig. 3A. Consistent with known function of SR1, the genes were highly enriched for the GO terms that are predominantly associated with plants response to pathogens/pests. The other highly enriched GO terms include abiotic stress and hormonal responses. One of the GO terms that is of special interest is “response to salt stress” for the following reasons: i) it is the second most enriched GO term after “response to bacterium” ii) this GO term comprises 27 genes (second most of all other categories), iii) expression of the majority of these genes is altered in opposite direction in the mutant and complemented plants (see section on salt stress below) and iv) SR1 was not previously known to be involved in salt stress.

GO analysis with the down-regulated genes revealed enrichment for only 37 GO terms (Additional File 5). The highest enrichment for biological processes is associated with photosynthesis (Fig. 3B). Importantly, unlike the GO terms observed in up-regulated DE genes, there was a significant enrichment for GO term associated with only cold stress. Interestingly, down-regulated DE genes with SR1 binding motif also contributed towards the process of “response to bacterium” (Fig. 3B). These results indicated that genes involved in a biological process can be either up- or down-regulated by SR1 depending on the gene.

### SR1 regulates the expression of other SRs

**Figure 4:**
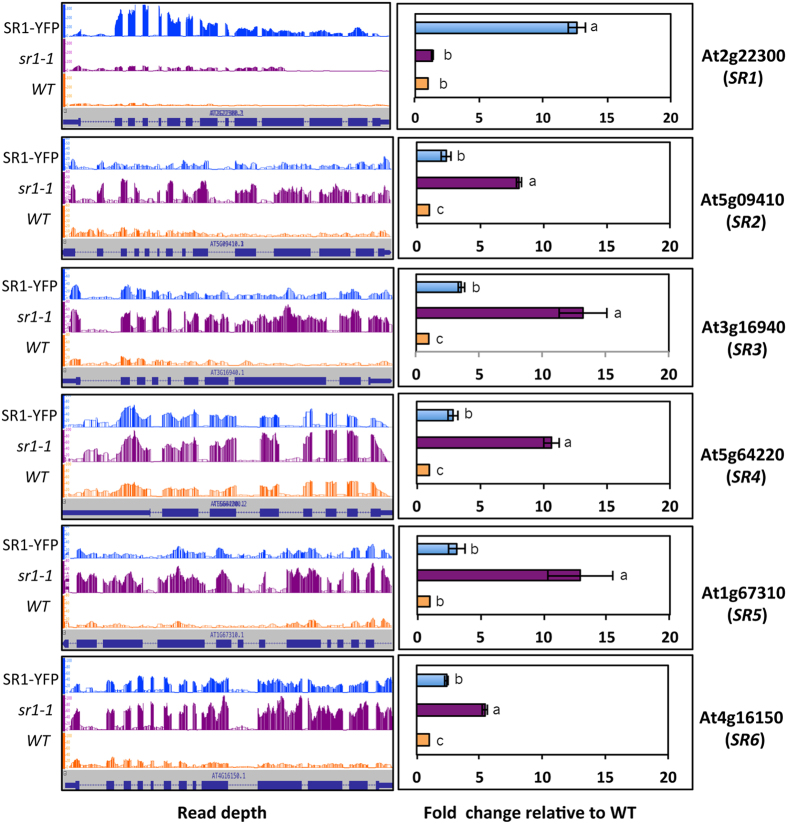
SR1 represses the expression of other members of the SR family. Expression profiles of *SRs* in WT, *sr1-1* and SR1-YFP lines. Panels on left show relative sequence read abundance as histograms (IGB view) in WT, *sr1-1* mutant and SR1-YFP. The Y-axis indicates read depth with the same scale for all three lines. The gene structure is shown below the read depth profile. The lines represent introns and the boxes represent exons. The thinner boxes represent 5′ and 3′ UTRs. Right panels show fold change in expression level relative to WT based on RT-qPCR analysis. WT values were considered as 1. Student t-test was performed and significant differences (P < 0.05) among samples are labeled with different letters. The error bars represent SD.

Analysis of promoters of six Arabidopsis *SR*s (*SR1 to SR6*) for the presence of SR1 binding motifs revealed that *SR3, SR4, SR5* and *SR6* contain one or more of these motifs (Supplementary Table S2), suggesting that their expression could be regulated by SR1. To test if any of these *SRs* are mis-regulated in *SR1* mutant, we checked RNA-seq data for their expression. Interestingly, the expression of all five *SR*s (*SR2* to *SR6)* is significantly elevated in the mutant and fully or partially suppressed in the complemented line (Fig. 4, left panel). To validate these RNA-seq results, RT-qPCR was performed and the results were in agreement with RNA-seq data (Fig. 4, right panel), indicating that SR1 suppresses the expression of other *SR*s.

### SR1 regulates expression of many transcription factors

The observed DE genes are likely due to direct and indirect effects of SR1; i.e., SR1 may directly bind the promoters of these genes and regulate their expression or regulate other TFs, which in turn regulate expression of down-stream genes. In Arabidopsis, there are over 1716 genes encoding TFs, which are grouped into 58 families^36^. Among the DE genes, we found 179 TFs belonging to 40 families (Supplementary Fig. S5 and Additional File 6). Of these families, WRKY (P < 0.0006), S1Fa like < P, 0.0007), GATA (P < 0.01), ERF (P < 0.03), EIL (P < 0.04) and ZF-HD (P < 0.04) are highly enriched (Supplementary Fig. S5A). Further examination of the TF families revealed that the genes of 33 of them contain SR1 binding sites (*VCGCGB* and *MCGTGT*) in their upstream region (−1000 bp of TSS) (Additional File 6), suggesting that they are likely direct targets of SR1. The number of TFs in each family that are affected and the direction of their expression change (up or down) in the mutant are shown in Supplementary Fig. S5B. Interestingly, expression of all TFs in certain families (e.g. WRKYs, NAC and GRAS) is up-regulated whereas all members in some other families are suppressed (e.g. ZF-HD, NF-Y3, Tri-helix and TALE) (see Supplementary Fig. S5B). The fact that expression of about 10% of all TFs is altered in the mutant suggests that many of the SR1-regulated genes in our DE list, especially those that do not contain SR1 binding motif, are likely indirect targets of SR1.

### SR1 negatively regulates salt stress tolerance

**Figure 5:**
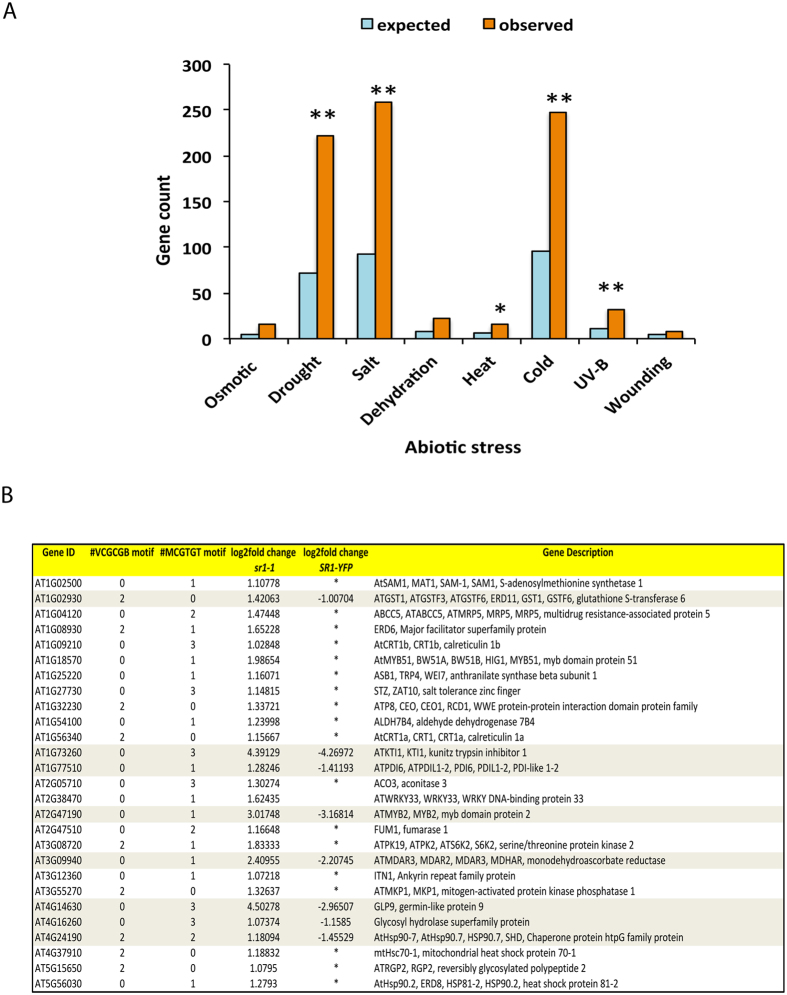
Abiotic stress responsive genes are over-represented in DE genes. (**A**) A significant number of DE genes are associated with abiotic stress response in comparison with genome background with a P < 0.0001 (**) and P < 0.05(*). (**B**) SR1 regulates the expression of salt-responsive genes. List of salt-responsive genes that are enriched in the GO term “response to salt stress” is presented. Transcript levels of these genes in the mutant and complemented line and the number of SR1 binding motifs in the upstream 1000 bp of the TSS are presented. Asterisks in the table indicate that the expression level in the complemented line is restored to wild type. In case of eight other genes that are highlighted, their expression is repressed in SR1-YFP as compared to the mutant.

**Figure 6:**
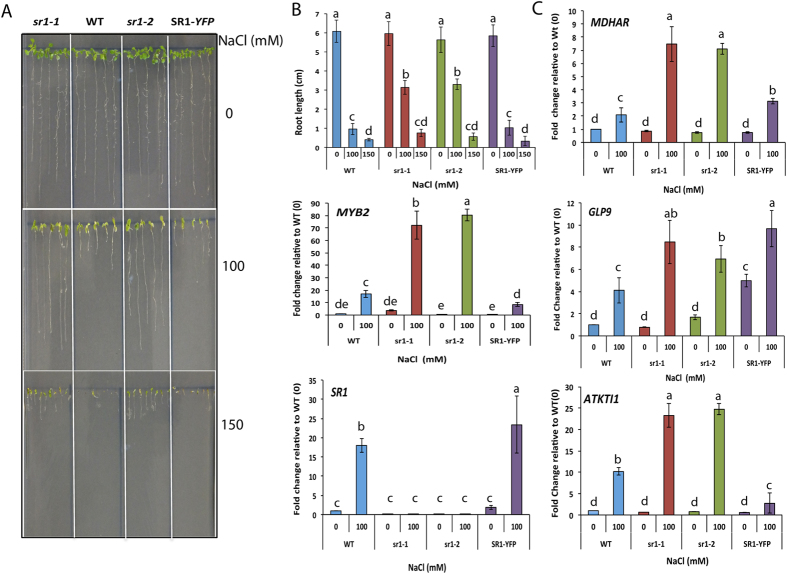
SR1 is a negative regulator of salt tolerance. (**A**) Growth of seedlings of WT, *sr1-1, sr1-2* and SR1-YFP on MS plates containing different concentrations of salt. Seeds were plated on ½ strength MS medium supplemented with 0, 100 and 150 mM of NaCl and were allowed to germinate and grow for two weeks. The photographs were taken after two weeks. (**B**) Top panel: root length was measured for each seedling for all four genotypes and plotted against the concentration of NaCl. Three biological replicates were used. Eight seedlings for each genotype per treatment for each biological replicate were included. Middle and Bottom panels: Expression levels of *MYB2* and *SR1* TFs under salt stress in different genotypes. Two-week-old seedlings grown on MS medium supplemented with 0 and 100 mM NaCl concentrations were used. A significant increase in the expression of these two TFs was observed. Salt-induced enhancement of *MYB2* expression level was significantly higher in *sr1-1* and *sr1-2* lines. (**C**) SR1 regulates the expression of other salt-responsive genes. Expression levels of *MDHAR*, *GLP9* and *ATKTI1* in two-weeks-old seedlings exposed to 0 and 100 mM NaCl are determined by RT-qPCR. The expression levels of salt-responsive genes were normalized with *ACTIN2*. Fold change in expression level relative to WT controls (WT-0) is presented. WT-0 values were considered as 1. Student t-test was performed and significant differences (P < 0.05) among samples are labeled with different letters. The error bars represent SD.

Since the promoters of a large number of DE genes contained RSRE, we performed enrichment analysis to determine if a particular stress responsive genes contributed maximally to the DE list. This analysis revealed a substantial enrichment (P < 0.001) of different abiotic stress responsive genes with a large number of them implicated in salt stress (Fig. 5A and Additional File 7). Interestingly, 27 salt-responsive genes are up-regulated in the mutant. Furthermore, in the complemented line expression of these genes was either restored to the wild type level or repressed (Fig. 5B). GO term enrichment analysis of SR1-binding motif containing up-regulated genes also showed strong enrichment of a term associated with salt stress (Fig. 3A). SR1 is known to regulate cold-induced gene expression^24^, but its function in salt stress is not known. We, therefore, investigated the role of SR1 in salt stress tolerance. Wild type, two homozygous loss-of-function mutants of *SR1* (*sr1-1* and *sr1-2)* and the complemented line^28^ were tested for salt tolerance. Root growth of all four genotypes was scored for salt tolerance by growing them on different concentrations (0, 100, 150 mM) of NaCl. Interestingly, a significant difference in the primary root length in a NaCl concentration dependent manner was observed (Fig. 6A). At 100 mM NaCl, a significant difference in root length was observed among the genotypes (Fig. 6A). A significant suppression in the primary root growth was noted in WT and SR1-YFP lines as compared to mutant lines (*sr1-1* or *sr1-2*), indicating decreased sensitivity of mutants to salt stress (Fig. 6A, middle panel) as compared to WT and SR1-YFP. Even at 150 mM NaCl, mutants were found to be more tolerant to salt stress. These results suggest that SR1 negatively regulates salt tolerance.

### SR1 suppresses the expression of salt-responsive genes

**Figure 7:**
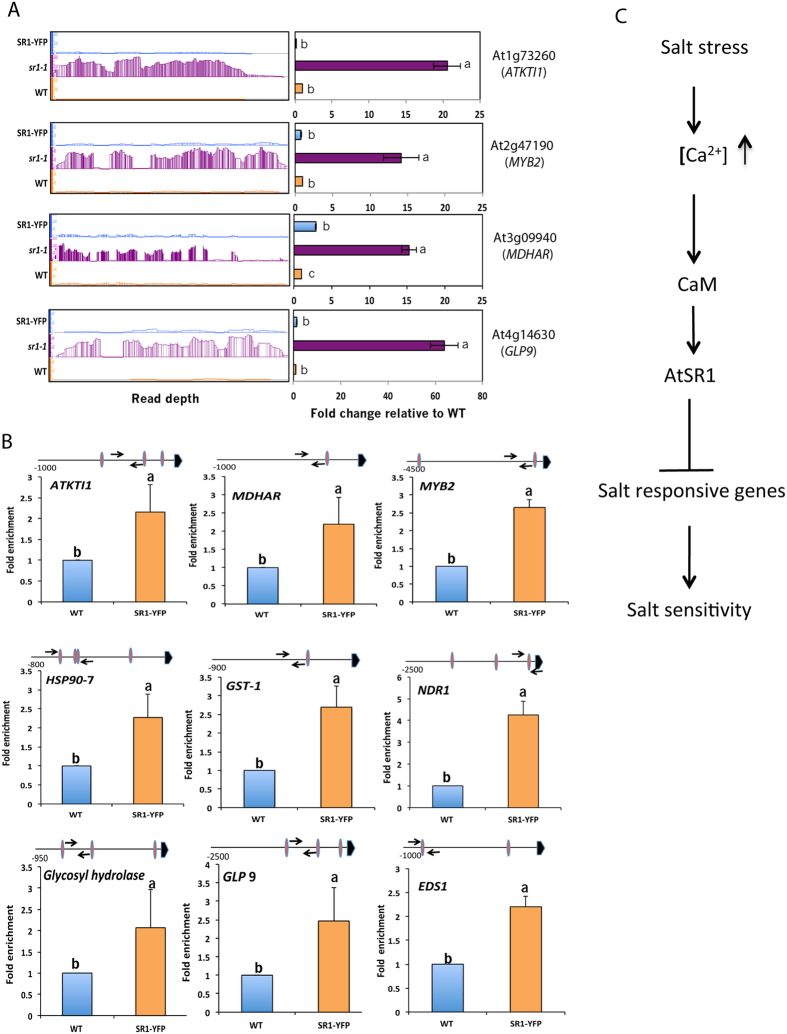
SR1 regulation of salt-responsive genes. (**A**) Expression levels of a few representative salt stress-responsive genes in WT, *sr1-1* and SR1-YFP. Left Panels: relative sequence read abundance (IGB view) as histograms in wild type (WT), *SR1* mutant (*sr1-1)* mutant and the complemented line (SR1-YFP). The Y-axis indicates read depth with the same scale for all three lines. Right panels: Expression analysis of salt-responsive genes using RT-qPCR. Panels on right show fold change in expression level relative to WT. WT values were considered as 1. Student t-test was performed and significant differences (P < 0.05) among samples are labeled with different letters. The error bars represent SD. (**B**) ChIP-PCR of upstream regions of salt-responsive genes containing *VCGCGB* or *MCGTGT* or *MCGCGT* + *VCGCGB.* Chromatin from 15-day-old seedlings from WT and SR1-YFP was immunoprecipitated with anti-GFP antibody and used in PCR with primers flanking the putative SR1 binding sites. The results obtained from four independent ChIP experiments were used to calculate fold enrichment. Data was normalized to DNA input levels as well as *ACTIN2*. The values of WT were considered as 1. Student t-test was performed and significant differences (P < 0.05) among samples are labeled with different letters. Schematic diagram over each panel shows SR1 binding sites (as oval shape) and the location of primers used in ChIP-PCR are indicated with arrows. Bold arrowhead indicates TSS. (**C**) Proposed model for the role of SR1 in salt stress response (see text for details).

To gain further insights into the role of SR1 in salt stress, the expression level of 27 salt-responsive genes under the GO category of “response to salt stimulus” was compared in *sr1-1* and SR1-YFP lines. Nineteen out of 27 salt-responsive genes were represented in both *sr1-1* and SR1-YFP data sets and their expression profiles were opposite to each other (Fig. 5B). Motif analysis of upstream regions of these genes indicated that a number of them contain SR1 binding motif (*VCGCGB* or *MCGTGT)* (Supplementary Fig. S6A). Orthologs of four Arabidopsis genes (At1g73260, At2g47190, At3g09940 and At4g14630) that were previously reported to be involved in salt tolerance^37^,^38^,^39^,^40^ and contain an SR1 binding motif in their promoter were selected as representatives to analyze their expression under control and salt stress conditions. The expression pattern of these four genes was verified by RT-qPCR analysis. Expression levels of all four genes were significantly higher in both loss-of-function mutants as compared to WT or SR1-YFP in the presence of salt (Fig. 6B, middle panel and Fig. 6C), suggesting that SR1 represses the expression of these salt-responsive genes. Analysis of RNA-seq data for expression of these four genes also showed increased expression in the mutant and their expression was restored to the wild type in the complemented line (Fig. 7A, left panel). The expression pattern of these four genes was confirmed by RT-qPCR analysis (Fig. 7A, right panel).

Majority of the salt-responsive genes are known to contain *cis*-elements in their promoter regions to which known TFs bind. These include G box (*CACGTG*), N box *CACG*[*G/A*]*C and NAC* (*CATGTG*) that bind G_box bHLH, N_box_bHLH and Nac_box_NAC TFs, respectively. To understand the regulation of these salt-responsive DE genes by SR1, POBO analysis was performed for the enrichment of these *cis*-elements as well as RSRE (*VCGCGB*) element in the upstream regions of all salt-responsive genes. A significant enrichment (P < 0.0001) for *VCGCGB* and *MCGTGT* was observed in the upstream region (−1000 bp) of the salt-responsive genes that were up-regulated (Supplementary Fig. S6A). In contrast, no enrichment for *MCGTGT* motif was noted in the upstream regions of down-regulated genes (Supplementary Fig. S6A). Further, significant enrichment for the G box (*CACGTG*), N box (*CACGGC*) and no enrichment for *NAC (CATGTG)* element in the promoter regions of the up-regulated salt stress-responsive genes were observed (Supplementary Fig. S6B). Significantly, enrichment of specific sequences (*ACGTGT*, *CCGTGT*, *ACGCGT*, and *ACGCGC*) within the SR1 binding consensus motif was also observed (Supplementary Fig. S6B). In contrast to the up-regulated salt-responsive genes, a significant enrichment for only G box (*CACGTG*) element and the SR1 binding motif *ACGTGT* was found in down-regulated salt-responsive genes (Supplementary Fig. S6C). These results clearly suggest dual regulation of salt responsive genes by different TFs and preferential usage of certain *cis*-elements (*ACGTGT*, *CCGTGT*, *ACGCGT*, and *ACGCGC*) within the consensus motif of these transcription factors.

### SR1 binds to the promoter regions of salt-responsive genes

Earlier studies have shown that SR1 binds to the promoter regions of *EDS1*, *NDR1* and *EIN3* that are involved in plant defense and ethylene signaling^28^,^30^. As shown in Supplementary Fig. S6A, there is a significant enrichment of SR1 binding sites in the DE genes that are responsive to salt stress. Consistent with this, expression levels of salt-responsive genes were significantly up-regulated in *sr1-1* (Fig. 5B). Restoration of transcript levels of salt-responsive genes in the SR1-YFP line to wild type level and the presence of SR1 binding sites in their promoter regions suggest that these are potential direct targets of SR1. To further confirm that SR1 regulates the salt-responsive genes, we determined the expression levels of four of these genes in WT, *sr1-1* and SR1-YFP using RT-qPCR. This analysis indicated significantly higher transcript levels of the salt-responsive genes in *sr1-1* (Fig. 7A, right panel). To confirm that SR1 binds to the promoter region of these genes, we performed ChIP-PCR assays using the complemented line expressing SR1-YFP. First, we confirmed whether there is a significant enrichment for the promoters of known targets of SR1 such as *EDS1* and *NDR1* in the ChIP’ed DNA obtained with anti-GFP antibody. A significant enrichment for the *EDS1* and *NDR1* promoters was observed thus validating earlier reports (Fig. 7B). We then performed enrichment analysis for promoters of several salt-responsive genes (*ATKTI1*, *MDAR3*, *HSP90*-7, *GST1*, *Glycosyl hydrolase GLP9* and *MYB*2) whose expression is increased in the mutant and contained one or more SR1 binding sites. Interestingly, a significant enrichment for promoters of these genes was noted in immunoprecipitated DNA (Fig. 7B), suggesting *in vivo* binding of SR1-YFP to these promoters and direct regulation of these genes by SR1. To address the specificity of SR1 binding to these promoters, we performed ChIP-PCR with primers corresponding to the promoter of *ACTIN2*, whose expression is not affected in the mutant (Supplementary Fig. S8) and also to two other genes [*GRAS2* (*At1g07530*) and *At1g15790*] that are misregulated in *sr1*, but do not contain SR1 binding motifs. For all three genes, there was no enrichment of promoters in the ChIP’ed DNA (Supplementary Fig. S8), indicating that binding of SR1 salt-responsive genes is specific.

## Discussion

### SR1 regulates expression of genes involved in multiple stress responses

Recent studies using *SR1* loss-of-function mutants have shown that it regulates biotic and cold stress responses^24^,^28^,^29^,^31^,^33^. Despite its important role in multiple stress responses, a comprehensive analysis on SR1-regulated genes is lacking. Our global transcriptome analysis using RNA-seq revealed that a large number of genes involved in diverse stress responses are regulated either directly or indirectly by SR1 (Figs 1 and 5). Previously Galon *et al.*^29^ compared the expression of genes in WT and *sr1-1* using microarrays and identified only 105 DE genes (99 up-regulated and 6 down-regulated genes)^29^. In that study, a complemented line was not included, hence it was difficult to ascertain that these DE genes are SR1-regulated. Our study significantly differs from the former study in a number of ways. Here we used next generation sequencing that significantly increased the depth of transcriptome analysis. More importantly, the use of a complemented line in which mutant phenotypes are rescued allowed us to identify the genes that are regulated specifically by SR1 (Supplementary Fig. S1). Our study revealed thirty times more DE genes as compared to the previous study^29^. This huge difference in the number of DE genes is likely due to the technology used here and the depth of RNA-seq. Over half of the DE genes reported in the previous study were found in our analysis. The absence of some DE genes from a previous study in our list could be due to limitations associated with different methodologies such as probe cross hybridization in microarray or more likely due to the tissues used for DE analysis as the age of the plants used in these two studies is different. In fact, developmental regulation of expression levels of *SRs* has been previously reported^12^,^13^,^23^. Reproducibility among replicates (Supplementary Fig. S2), full or partial restoration of expression of ~85% of DE genes in our complemented line to wild type level (Supplementary Figs S2 and S3) and RT-qPCR validation of expression of a number of randomly selected DE genes indicates that the DE genes are bona fide SR1 targets. Enrichment of DE genes in multiple abiotic stress-responses indicates that SR1 plays a major role in cross-talk between multiple stress signal transduction pathways (Fig. 5 and Additional File 7). Earlier, SRs were shown to differentially respond to various stresses such as heat, cold, salinity, drought, UV and stress hormones such as ethylene and ABA^18^. Further, many of the SRs have been implicated for their regulatory role in abiotic stress responses^19^,^20^,^26^,^27^,^33^. GO analysis of the DE genes indicated high enrichment of GO terms associated with diverse cellular processes that are critical for plant responses to biotic stresses such as bacteria and fungi, and abiotic stresses including drought, cold, salt and oxidative stress. Response to hormones such as ABA and auxin was also observed (Supplementary Fig. S7 and Additional File 8). These results suggest that SR1 could function as an important integrator of variety of stress responses. Consistent with these results, SR1 is already known to play an important role in at least four different stress responses^24^,^28^,^29^,^31^,^33^.

### SR1 binding motifs containing genes are both up- and down-regulated

Earlier studies identified *CGCG* and *CGTG* as core sequences to which SR1 binds through its CG1 DNA binding domain^23^. Furthermore, several studies identified *VCGCGB* and *MCGTGT* as consensus element, through which the SR1 regulates the expression of target genes^24^,^28^,^33^,^35^. Analysis of DE genes showed that >59% of SR1-regulated genes contain *VCGCGB and MCGTGT* elements and these motifs are significantly enriched in their promoter regions (Fig. 2). Among the genes that contain SR1 binding motif, in up-regulated genes both elements contributed towards the enrichment whereas highest representation of *MCGTGT* motif was observed in the down-regulated genes. Further, POBO analysis using the whole genome as a background also confirmed this observation (Fig. 2). The up-regulated genes containing SR1 binding sites, not only highly enriched for GO terms related to defense response to bacterium and fungi, but also for response to salt stress, water deprivation, and response to some hormones. In contrast, GO term enrichment of down-regulated genes that contain SR1 binding motifs exhibited significant enrichment for “response to cold” and “cold acclimation” apart from other cellular processes. This is consistent with the previous reports where SR1 was shown to function as a positive regulator of genes involved in the cold response^24^,^33^. Indeed, a preferential enrichment of either up- or down-regulated SR1 binding motif-containing genes for a biological process indicates that SR1 binds different *cis*-elements for regulation of different biological processes. Further, it was proposed that SR1 induced activation of the CBF2 is mediated through *VCGCGB* element in the promoter^24^.

Previous studies using loss- or gain-of-function alleles of *SR1* have shown that it acts as a critical regulator of both basal and systemic acquired resistance^28^,^33^,^41^. A significant increase in the basal levels of SA in the loss-of-function mutants of *SR1* has been reported^24^,^28^,^33^. Our gene expression analysis also indicated that 66% of the SA responsive genes have *VCGCGB or MCGCG* elements in their promoters indicating that they are potential direct targets of SR1. Some of these include *TGA3*, *NAC0062*, *CBP60G*, *EDS5*, *WRKY8*, and *MPK1.* Earlier *CBP60g* along with *SARD1* had been described as key regulators of *ICS1* induction and SA synthesis^42^,^43^. It has been suggested that SR1 may regulate the defense response through binding to the promoters of the genes or through activation of other repressor proteins^28^,^29^. In fact, Du *et al.*^28^ have shown direct binding of SR1 to the *EDS1* promoter and repression of its expression, indicating repressive activity of this TF in regulating these genes.

### SR1 suppresses the expression of other members of SR family

Loss-of-function of *SR1* significantly relieved the suppressive effect of SR1 on other *SRs* expression. Furthermore, expression of other *SRs* is significantly reduced in the complemented line (Fig. 4), indicating that SR1 controls the expression of other *SR* genes. Regulation of expression of some of these *SR* genes is likely through direct binding of SR1 to *cis* elements (*VCGCGB* or *MCGTGT*) in their promoter. With the exception of *SR2*, promoters of the rest of the *SRs* (*SR3, 4, 5* and *6*) do contain the *cis*-elements variation of the *CGCG* box, which could be involved in regulation of these genes by SR1 (Supplementary Table S2). We analyzed the promoter sequence of *SRs* for non-canonical binding motifs (i.e. with core sequence being similar and nucleotide at 5′ and 3′ end of the element being different) and found them to contain motifs related to SR1 binding sites (Supplementary Table S2). Interestingly, elevated expression level of *SR2* in *sr1-1* and its down-regulation in the complemented line, even in the absence of SR1 binding motifs in its promoter region, indicate the existence of an alternate mechanism by which SR1 regulates *SR2* expression. Previously, the *VSP1* promoter, which does not contain a canonical SR1 binding motif, was shown to be regulated directly by SR1^32^, thus indicating the existence of alternate regulatory pathways. Thus, it is possible that SR1 also regulates *SRs* through non-canonical *cis*-elements in their promoters.

### Indirect regulation of SR1-regulated genes

Enrichment analysis for TF families in DE genes indicated highest enrichment for genes in the WRKY, EIL, ERF, ZF-HD and S1Fa TF families. The WRKY TFs, which were all up-regulated (Supplementary Fig. S5A), bind W-box in the defense genes that are primarily implicated in regulation of defense responses against pathogen infection. However, these TFs are also implicated in other cellular processes such as abiotic stresses^44^. Some members of this family (*WRKY18*, *WRKY33*, *WRKY40*, *WRKY46*, *WRKY70*, *WRKY53*, *WRKY70* and *WRKY75*) have SR1 binding sites in their promoter (Additional File 6), indicating that they are likely direct targets of SR1. Given that GO terms enrichment for the “response to bacterium/pathogen” and “response to abiotic stresses” was observed in DE genes, it is possible that these TFs may regulate the expression of DE genes that do not contain an SR1 binding motif. In the down-regulated genes, the highest representation of ZF-HD, ERF, AP2, bHLH, and TCP TF families was observed, indicating that the members of these families are positively regulated in SR1. Together, these data indicate a complex network of regulation of expression of TFs by SR1.

### RSRE is enriched only in up-regulated genes

Recent studies identified *VCGCGB* as the core element that is enriched in a majority of early-activated genes that are also regulated by Ca^2+^ under stress conditions^25^. As the RSRE element *VCGCGB* is identical to the binding site of SRs (*VCGCGB*), many studies implicated SRs in general and SR1 in particular in regulation of general stress responses. Our analysis of the promoter region of all the DE genes indicated that a significant percentage of the genes contain this element, thus establishing their role in general stress response (Fig. 2). Further, the fact that the majority of these genes are misregulated in *sr1-1* and are implicated in various stress signaling pathways, confirmed the significant role played by SR1 in their regulation. Interestingly, POBO analysis indicated the enrichment of RSRE motif only in the promoter regions of the DE genes that were up-regulated, but not in genes that were down-regulated (Fig. 2C). This might be due to increased occurrence of abiotic stress responsive (with exception of cold responsive) genes in up-regulated genes and/or that the negative regulation by SR1 is not mediated through the RSRE element. As evident from Fig. 5A, significant enrichment of GO terms for the abiotic stresses such as “responses to salt stress” and “water deprivation” was observed only in the up-regulated DE genes. Furthermore, enrichment of *VCGCGB* motif was significantly higher in the up-regulated DE genes. Absence of enrichment for *VCGCGB* in the down-regulated DE genes and enrichment for GO terms “response to cold” and “cold acclimation” clearly suggest that SR1 positively regulates cold responsive genes through utilization of *VCGTGT* rather than *VCGCGB* (Figs 2C and 3, and Supplementary Fig. S6A). In fact, a significant enrichment of the SR1 binding sites, *VCGTGT* and *VCGCGB*, was noted in the early cold-responsive genes^33^.

### SR1 confers salt sensitivity by repressing the expression of salt-responsive genes

GO analysis of the up-regulated genes that contain SR1 binding sites in their promoters exhibited significant enrichment of a GO term associated with “response to salt stress (Fig. 3A)”, suggesting a new role for SR1 in salt tolerance. Interestingly, both mutant lines of *SR1* performed better in terms of root growth under increasing concentrations of NaCl when compared with the WT and SR1-YFP seedlings. Thus, our results suggest that SR1 acts as a negative regulator of seedling growth under salt stress. This negative regulation of salt stress by SR1 is similar to that observed under biotic stress^28^,^29^ and differs from that of the cold stress response^24^, where it functions as a positive regulator. Previously, Galon *et al.*^19^ and Pandey *et al.*^20^ identified SR2/CAMTA1, another member of SR family TF, to be a positive regulator of salt stress as mutants lacking this TF exhibited increased sensitivity to salt stress and drought stress, suggesting that SR1 and SR2 have opposing functions in salt stress.

In order to resolve the regulation (direct versus indirect) by SR1, salt-responsive genes were identified and subjected to POBO analysis for enrichment of *VCGCGB* in their upstream region. Analysis of the promoters of the salt-responsive DE genes revealed significant enrichment for RSRE (*VCGCGB*) in up-regulated genes (Supplementary Fig. S6A). Hence, it is possible that some of these genes could be direct targets of SR1. Similar analysis of promoters of down-regulated salt-responsive genes did not show enrichment of RSRE, suggesting that i) SR1 utilizes different motifs to regulate expression of these genes and/or ii) other proteins (including other SRs) might activate these genes, whose expression may be regulated by SR1.

As our data showed a negative regulatory role for SR1 in salt stress, we determined the effect of SR1 mutation on the expression levels of the genes associated with the biological process “response to salt stress”. Twenty-seven genes associated with this GO term were screened for the presence of SR1 binding sites in their promoters, their expression levels and ability of SR1 to complement their expression in SR1-YFP line. Although several genes fit these criteria (Fig. 5B), *KTI1*, *MYB2*, *MDAR3*, *GLP9* were selected along with *SR1* and their expression levels were determined in different genotypes in response to salt stress. Earlier reports have shown that overexpression of *MYB2* and orthologs of other three genes confer salt tolerance^38^,^39^,^45^,^46^. Exposure to salt stress significantly enhanced their expression levels by two-fold in both WT and SR1-YFP seedlings. In contrast, >12 to 15 fold higher induction of these genes was observed in both mutant alleles of *SR1*. Interestingly, *SR1* expression in WT and SR1-YFP was about 12 to 16 fold higher in salt-treated seedlings as compared to their respective controls (Fig. 6). Since the 35S promoter driving *SR1-YFP* is known to be non-responsive to salt stress, the observed increase in *SR1-YFP* transcript may be due to its increased stability in the presence of salt^47^.

Many members of different TF families are known to regulate expression of genes involved in salt stress by binding to the various *cis*-elements in the promoters of salt-responsive genes^37^,^38^. In response to salt stress, TFs such as G_box_BHLH and N_box_bHLH bind to the *cis*-element *CACGTG* and *CACG*[*G/A*]*C*, respectively, and regulate their expression^48^,^49^. In this study, we analyzed the enrichment for *cis*-elements to which various TFs bind in the upstream regions of salt-responsive DE genes. We compared if there are any differences in the enrichment pattern among the salt-responsive up- and down-regulated DE genes using POBO analysis. We found enrichment (P < 0.0001) for G Box (*CACGTG*), N box (*CACGGC*) but not NAC (*CATGTG*) in up-regulated salt-responsive genes (Supplementary Fig. S6B). Only G Box (*CACGTG*) enrichment was noted in the down-regulated genes (Supplementary Fig. S6C). Analysis for co-enrichment of SR1 binding motifs showed enrichment for *ACGTGT, CCGTGT, ACGCGT* and *ACGCGC* in the promoter regions of both up- and down-regulated salt-responsive genes. The observations that i) promoter regions of these genes have 1 to 3 SR1 binding sites, and ii) SR1 binds to promoters of some of these genes (Fig. 7B) provide evidence that SR1 directly regulates their expression.

Based on our work we propose a model (Fig. 7C) to explain the role of SR1 in salt stress response. Previous studies have demonstrated that exposure of plants to salt stress changes cytosolic Ca^2+^ levels^50^. In addition, Ca^2+^ through CAM has been shown to regulate SR1 activity^28^. Our work showed that AtSR1 either directly and/or indirectly suppresses the expression of salt-responsive genes that are necessary for salt tolerance there by conferring salt sensitivity. In summary, our results showed that a large number of genes that are associated with biotic and abiotic stress responses are regulated by SR1. A large fraction of these genes (~59%) contain one or more binding sites of SR1 in their promoter region, suggesting that they may be regulated directly by this TF. Our transcriptome analysis revealed a novel role for SR1 in salt stress. By analyzing growth phenotypes and salt-responsive genes we confirmed that SR1 functions in salt stress response. Further, our results showed that SR1 functions as a negative regulator of salt tolerance. These results provide novel insights into the role of SR1 in abiotic stress tolerance in general and salt stress in particular. Future studies using chromatin immunoprecipitation followed by high-throughput sequencing (ChIP-seq) should allow identification of direct targets of SR1^51^.

## Methods

### Plant Materials

Three Arabidopsis genotypes - WT (Columbia-0), two alleles of *SR1* mutant (*sr1-1*, *sr1-2)* in Col-0 background, and a complemented line (SR1-YFP) – used here were developed earlier^28^. Surface sterilized seeds were sown in sterilized soil, allowed to germinate and grown for 40 days in a growth chamber at 21 ± 1 °C with 60% humidity, 200 μmoles/m^2^/sec light under day neutral condition. To test salt stress tolerance in these genotypes, surface sterilized seeds were plated on ~70 ml of ½ strength MS medium supplemented with 1% sucrose, 0.5 g/L of MES along with 0, 100 or 150 mM NaCl and 0.8% (w/v) Phytoblend in square sterile Petri dishes. The seeds were germinated and seedlings were grown vertically for two weeks to score for the seedling growth and root length. All genotypes were grown on the same plate to minimize the differences due to any changes in microenvironment. After 14 days, root length was measured and seedlings were photographed. All experiments were performed three times with a minimum of three replicates.

### Western blot analysis

Leaf material was flash frozen, ground in liquid nitrogen and nuclear extracts were prepared from nuclei preparation essentially as described in Xing *et al.*^52^ with slight modifications. The pellet containing nuclei was resuspended in nuclear lysis buffer and sonicated using Covaris M220 Focused –ultrasonicator for 8 min at 7 °C with settings of peak power 75, duty factor 5 and 200 cycles/burst. The extract was clarified by centrifugation for 10 min at 16,000 g at 4 °C. Immunoprecipitation was performed essentially as described in Xing *et al.*^52^ using Chromotek GFP-TRAP_A beads. Immunoprecipitated protein was separated from beads by boiling at 95 °C for 10 min in 60 μl of 1× SDS loading buffer. Thirty μl of extract was resolved in 12% SDS gels and blotted on to a PVDF membrane. The blot was probed with anti-GFP antibody (sc-8334, Santa Cruz Biotechnology) and detected with secondary antibody conjugated with alkaline phosphatase detection system.

### RNA–seq

Total RNA from leaves (collected at 4 p.m.) of 40-day-old plants of three genotypes was isolated using miRNAeasy kit (Qiagen, USA#217004). Any contaminating genomic DNA was removed using on column DNAse digestion. Ribosomal RNA was removed using a Ribozero Plant kit and the sequencing libraries were prepared from rRNA-depleted samples using TruSeq stranded RNA-seq kit (Illumina) as per manufacturer instructions and single-end sequencing of the library was done at the Genome Sequencing & Analysis Core Resource, Duke University using Illumina Hi seq 2000. All RNA-seq reads were deposited at NCBI in the GenBank sequence read archive (SRA) under the accession number SRP073518.

### Mapping of the reads and identification of DE genes

The reads were aligned to the TAIR 10 version of the Arabidopsis genome using TopHat^53^ using default settings. The read alignments were assembled into transcriptome assembly using Cufflinks. The assemblies for each replicate were merged together using Cuffmerge utility^53^. Using Cuffdiff tool^53^ the aligned reads and merged assembly for each genotype were utilized for calculating the expression level differences of various genes. The DE genes list was computed using Cuffdiff^53^. Those genes that met the following criteria were considered as DE genes: i) The q-value < 0.05, ii) the fold change >2, and iii) The sum of the RPKM from the comparing genotypes >10. The common genes that are represented in one or more data sets were identified using the VENNY (http://bioinfogp.cnb.csic.es/tools/venny/) a web-based tool. Heat map of differentially expressed genes was generated using CummeRbund^53^. Box-and-whisker plots of DE genes were generated using the log2 transformed expression values in WT, *sr1-1* and SR1-YFP with JMP Pro12 statistical software. For scatterplot analysis, the RPKM values were log2 transformed and genes with ≥1 value were used.

### Bioinformatics analysis to identify SR1 binding motif-containing genes

To identify the number of DE genes with SR1 binding motifs *VCGCGB* and *MCGTGT* in their promoter, “Patmatch” (Version 1.1) utility tool (www. arabidopsis.org) was used. This tool identifies the motif on both the strands from the dataset of “*TAIR10 Loci Upstream sequences-1000* *bp*”. 1000 bp sequence preceding the TSS was used for this analysis. Up- and down-regulated genes were included as input for scoring both type and number of SR1 binding motifs.

### GO enrichment analysis

GO analysis was performed for term enrichment using GeneCodis^54^. Single enrichment analysis with TAIR GO annotations was performed using the hyper geometric test with Benjamin-Hochberg FDR correction with a significance of P < 0.05. The genes that are up- or down-regulated for each data set were analyzed separately.

To identify various TFs in the DE genes, a list of all TFs was obtained from Plant TF Database (version 3.0)^36^ and all DE genes were queried against the total TF list. TAIR 10 ID of all TF genes was used as input for identifying the genes encoding the TF and classifying them based on the similarity with Total TF family list. The TFs and the genes responsive to various abiotic stress conditions were obtained from STIFB (Stress Responsive TF Database) (http://caps.ncbs.res.in/stifdb2/). Promoters of the genes that contained *cis*-element for binding of the TFs that are implicated in abiotic stress response were retrieved for the analysis. DE genes were queried against the list of the genes for a specific abiotic stress. Further, on the basis of overlap of locus ID (TAIR ID) between the lists of genes, they were further categorized into different subsets.

For the promoter analysis either 500 or 1000 bp upstream of the start codon was extracted from TAIR using an online tool for bulk sequence retrieval. For the estimation of the enrichment for a particular *cis*-elements in the set of promoter sequences (−500 or −1000 bp) were used as input for POBO analysis (http://ekhidna.biocenter.helsinki.fi/poxo/pobo)^55^.

### Validation of DE genes using RT-qPCR analysis

Primers for validation of DE genes using Real time qPCR (RT-qPCR) were designed using Primer Quest web tool (http://www.idtdna.com/Primerquest/Home/Index) from IDT (USA) (Supplementary Table S3). Nine DE genes were randomly selected and analyzed for their expression levels using RT-qPCR. cDNA from 40-day-old plants was prepared with SuperScript III first Strand Synthesis kit (Invitrogen), and diluted to 1:5 ratio with sterile nuclease free water, 1.5 μl of the diluted cDNA was used for each reaction. For every qPCR reaction, 5 μl of 2X LightCycler 480 SYBR Green I Master mix (Roche) was used along with 1 μl of 5 μM of each primer in a final reaction volume of 10 μl. For each genotype, cDNA from two independent biological replicates was used. Three technical replicates were used for each sample. RT-qPCR was performed in a Roche LC480 machine (Roche) using the preprogramed “SYBR green-I 96 well program”. *ACTIN2* was used as a reference gene as this gene does not exhibit any difference in its expression levels among the various genotypes (Supplementary Fig. S8). Fold change in expression was calculated and plotted with respect to WT. The expression level in WT for each gene is considered as 1.

### RT-qPCR analysis of salt-responsive genes

Fourteen-day-old control and salt-treated seedlings of different genotypes were collected and flash frozen in liquid nitrogen. The frozen tissues were ground to fine powder in 2 ml microfuge tubes with metal ball bearings. Total RNA was isolated using Trizol and then subjected to DNAse (Promega) treatment to remove any genomic DNA. Two μg of total RNA was used for cDNA synthesis using Superscript II reverse transcriptase (Invitrogen) as per manufacturer instructions. The cDNA was diluted 5 times and 2.5 μl/reaction was used as a template. Expression analysis was performed using RT-qPCR as described above. The data obtained was normalized with *ACTIN2* and fold change in the expression level was calculated relative to WT control i.e, 0 mM NaCl. The expression level in WT control was considered as 1. A minimum of three technical replicates and three biological replicates were used for each experiment.

RNA isolated from three genotypes was used for cDNA synthesis to analyze the expression of other members of SR family (*SR2-SR6)*. cDNA synthesis, primer design and RT-qPCR analysis were done as described above. The expression levels of the SR genes were normalized with *ACTIN2* and fold change in the expression was calculated relative to WT. The values of WT were considered as 1.

### ChIP-PCR

For chromatin immunoprecipitation (ChIP) assays, 15 day-old seedlings of WT and SR1-YFP were grown on ½ MS medium with 1% sucrose under 16/8 h day/night cycle at 21 °C. ChIP assay was performed as described by Werner Aufsatz with modifications using GFP-Trap_A beads (http://www.abcam.com/protocols/chip-using-plant-samples—arabidopsis). Briefly, nuclear extract was prepared from formaldehyde cross-linked (1%) seedlings of WT and SR1-YFP as above and diluted with ChIP dilution buffer and pre-cleared with bab-20 agarose beads. The pre-cleared nuclear extract was further incubated with GFP–Trap_A beads for 15 h at 4 °C on rotatory wheel. The beads were collected by centrifugation and washed sequentially with 1 ml of low salt wash buffer, 1 ml of high salt wash buffer, 1 ml LiCl wash buffer and 1 ml TE buffer. Each wash was carried out by resuspending the beads in wash buffer and rotating on a wheel at 4 °C for 5 min and centrifuging at 2500 g for 2 min, and the supernatant was discarded. The protein-DNA complex was eluted twice with 250 μl of elution buffer (1% SDS and 0.1 M NaHCO_3_) and reverse cross-linked by incubating the eluate at 65 °C for 6–8 h followed by 3 h of proteinase K treatment at 45 °C with gentle shaking. The DNA was purified using phenol:chloroform/isoamyl alcohol and precipitated using absolute ethanol followed by washing with 75% ethanol. Air-dried DNA pellet was resuspended in 70 μl of TE buffer with RNAse A (10 μg/ml). The precipitated DNA was used for qPCR with the primers specific to a region of promoter in the target genes. Data was normalized to DNA input levels as well as *ACTIN2*. The results obtained from four independent ChIP experiments were used to calculate fold enrichment. The values of WT were considered as 1.

## Additional Information

**How to cite this article**: Prasad, K. V. S. K. *et al.* Global gene expression analysis using RNA-seq uncovered a new role for SR1/CAMTA3 transcription factor in salt stress. *Sci. Rep.* **6**, 27021; doi: 10.1038/srep27021 (2016).

## Supplementary Material

Supplementary Information

Supplementary Dataset 1

Supplementary Dataset 2

Supplementary Dataset 3

Supplementary Dataset 4

Supplementary Dataset 5

Supplementary Dataset 6

Supplementary Dataset 7

Supplementary Dataset 8
