# Supplementary material for: Global gene expression analysis using RNA-seq uncovered a new role for SR1/CAMTA3 transcription factor in salt stress
Source: Sci Rep. 2016 Jun 2;6:27021. doi: 10.1038/srep27021 (PMC4890006; doi:10.1038/srep27021)
Supplement: Supplementary Information [file srep27021-s1.pdf]

Supplementary Tables and Figures

Global gene expression analysis using RNA-seq uncovered a new role for SR1/CAMTA3 transcription factor in salt stress

Kasavajhala V.S.K. Prasad[1], Amira A.E. Abdel-Hameed[1], Denghui Xing, and Anireddy S.N. Reddy*

Department of Biology and Program in Molecular Plant Biology, Colorado State University, Fort Collins, CO, 80523

[1]These authors contributed equally

Supplementary Table S1: Mapping statistics of RNA-seq reads

Sample	Total Reads	Reads mapped	Percent Mapped	Uniquely Mapped	Percent Uniquely Mapped	Multiple Hits
WTSR1_R1	37857222	35886091	94.8	32436144	90.4	3449947
WTSR1_R2	43257556	40792316	94.3	37269214	91.4	3523102
KOSR1_R1	45443405	42610742	93.8	39268747	92.2	3341995
KOSR1_R2	41943988	39615994	94.4	36355172	91.8	3260822
SR1YFP_R1	42479202	39987615	94.1	36689779	91.8	3297836
SR1YFP_R2	37006004	35056382	94.7	32469773	92.6	2586609

Supplementary Table S2. SR1 binding motifs in other *SRs* promoters

Promoter	Motif	Location
SR1/*CAMTA3*	*ACGTGA*	-365
	TCGTGT	-967
SR2/*CAMTA1*	------	-----
SR3/*CAMTA6*	CCGCGG	-748
	CCGCGA	-1348
SR4/*CAMTA2*	*ACGCGC*	-616
	CCGCGG	-1951
	ACGTGT	-302, -310
	ACGTGG	-593
	ACGTGA	-479
SR5/*CAMTA4*	*CCGCGG*	-1872
	ACGTGG	-35
	ACGTGC	-208
SR6/*CAMTA5*	*CCGCGG*	-1054, -1197
	ACGCGG	-541
	ACGTGT	-3391

Motif highlighted is consensus-binding motif of SR1. The motifs that are not highlighted contain a part of consensus motif.

Supplementary Table S3. Primers used in the current study.

S.No.	Name	Sequence
1	AtSR1-FW	CCA TTT AAA TAT GGC GGA AGC AAG ACG ATT CAG CCC A
2	AtSR1-RW	CGCGGATCCTTAACTGGTCCACAAAG ATGAGGACATA
3	Q-SR1-FW	CTCGGGAGGAGACTGAAATTG
4	Q-SR1-RW	AGGAGCAACACATTGGAGAATA
5	Q-SR2-FW	GGG TAT GAC TGG GCC ATT AAA
6	Q-SR2-RW	TTT CCT CCC TGC CAC TAA AC
7	Q-SR3-FW	CTC TGT GCC AGT CTT GGA TAC
8	Q-SR3-RW	GAG CAG TCC ACC CTT GTT TAT
9	Q-SR4-FW	CCA ATC TTA GCA GCA GGA GTT A
10	Q-SR4-RW	GAC AAG TAC AGC GAC AGT ATC C
11	Q-SR5-FW	TGG ATT GCA GGA AGA CTC AAA
12	Q-SR5-RW	GGA GCT ACC AGT GCA GAA TAA G
13	Q-SR6-FW	GGG ACC ATC TCT TTG AGC TTA C
14	Q-SR6-RW	CTC CAA GCC CTT TAG AGT CAT ATT
15	AT5G45890-FW	ATGAGGATGTCCCGGTTAATG
16	AT5G45890-RW	GTGAACACACCAGACGAATAGA
17	AT2G41850-FW	CCGGTACAGACAATGGAGTAAG
18	AT2G41850-RW	TTGCTCTTGTCGCAGTAGTC
19	AT3G01420-FW	ACGTCGACTTAGCTGCTTTAG
20	AT3G01420-RW	CTCCGTTAGATCTTCCCACTTG
21	AT3G60140-FW	GACAACGACGACGGTACAAA
22	AT3G60140-RW	CTCTTACGTCACACCCATCTTC
23	AT2G45220-FW	GAAGATCCGACCCGAATCAAA
24	AT2G45220-RW	GTCTCCAAGGTCTACCCAAATAAG
25	AT5G38710-FW	GCCTCAAATCCGTGTGTCTTAG
26	AT5G38710-RW	CCGACCAATACGCCATGTAATC
27	AT2G42540-FW	CTCAGTTCGTCGTCGTTTCT
28	AT2G42540-RW	GTTGAGGTCATCGAGGATGTT
29	AT5G15960-FW	GCTGAGGAGAAGAGCAATGT
30	AT5G15960-RW	CCGCATCCGATACACTCTTT
31	AT1G14250-FW	CAGTCACAGTTTCCTCGACTT
32	AT1G14250-RW	GGGTCTTCAACTATTCCATCCC
33	NDR1-CHIP-FW**	TTGGTTCTTTTTGATAACCCAAAGT
34	NDR1-CHIP-RW**	TTTGGTTTGCTGATTGGTTGATATT
35	EDS1-CHIP-FW**	TGGTTATGCAATTTGGTTTAGCCAA
36	EDS1-CHIP-RW**	ACCGAATTAACTAACTACACCTTCTT
37	ACTIN2CHIP-FW**	GATCCTAGTCTTTTAGTGTGCATTC
38	ACTIN2CHIP-RW**	ATTAAATGATTGATCGGTTTTCGTG
39	ATKTI1-CHIP FW	TTGTAATTTGTCAGGAACGGAGA
40	ATKTI1-CHIP RW	GTGTCCTGACGTGTGGATTT
41	MDAHAR-CHIP FW	TCACGAACGTTATCCCACTAAA
42	MDAHAR-CHIP RW	CATTGGCATTATTCACTCGAATCT
43	HSP90-7-CHIP-FW	TCTCTGGTGAGGAAGGAAGT
44	HSP90-7-CHIP-RW	CACTCATCCGTAGTAGCAATATGT

45	GST1-CHIP FW	TGATCTAACTCGAGCATCCAAC
46	GST1-CHIP RW	CCACAAGAATAGTCCTTCATCTACTA
47	Glycos transf-CHIP FW	ATACGGCTGCTCTTGTTAAGT
48	Glycos transf-CHIP RW	CCACTCATGAATTGGTTACTGATTT
49	GLP9-CHIP FW	AAGTAGTAACAGCCTCTCTCTTTC
50	GLP9-CHIP RW	TTGGGTTGCTTGATTCGTTAAG
51	MYB2-CHIP FW	CGTGATTGCACACAACAAGAAG
52	MYB2-CHIP RW	CACACAGTATCGCAGACGTAAG
53	AT1G15790 FW	TGTTCCAAATCTTGGGCTACAA
54	AT1G15790 RW	CCTCTTCCACAGTCAACAACTC
55	AT1G07530 FW	GGA CCT TACTGGCTTCGTTATG
56	AT1G07530 RW	GGGAGAGATGGTTTGGACTTTG
57	ACTIN2 FW	GGCAAGTCATCACGATTGG
58	ACTIN2 RW	CAGCTTCCATTCCCACAAAC
59	LBa1	TGGTTCACGTAGTGGGCCATCG

** Primers are adopted from Nie et al., 2012.

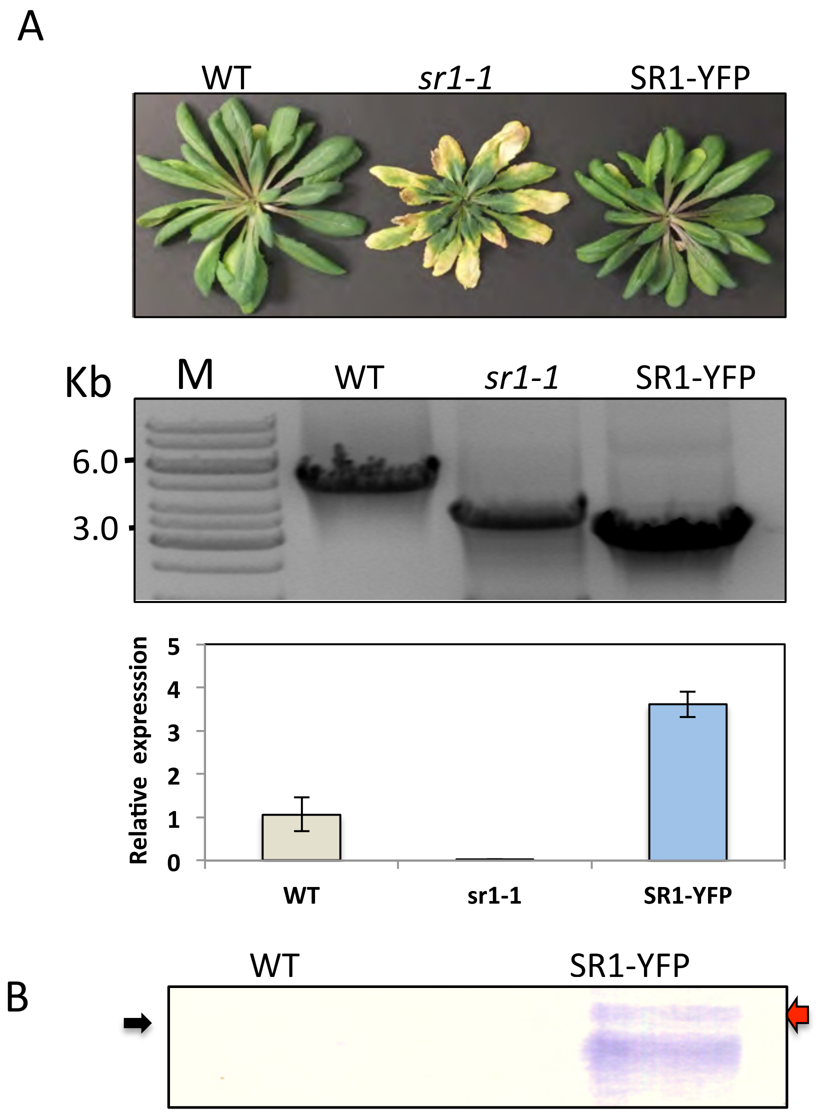

Supplementary Figure S1. Verification of genotypes used for RNA-seq. A) Top panel: Phenotypes of 40-day-old-plants of wild type (WT), *SR1* mutant (*sr1-1*) and *sr1-1* complemented with *SR1* (SRI-YFP) grown as described in the Methods section. Middle panel: Genomic PCR of the three genotypes. Prior to RNA-seq, genomic PCR was performed with *SR1*-specific primers in case of wild-type and SR1-YFP whereas SR1-specific forward primer and Lba1 reverse primer (T-DNA specific primer) were used for *sr1-1*. In all three cases the expected amplicon size was obtained. Bottom panel: Analysis of *SR1* expression using RT-qPCR in two-week-old seedlings of wild type (WT), *SR1* mutant (*sr1-1*) and complemented line (SRI-YFP). **B)** Immunodetection of SR1-YFP protein in the nuclear extracts of the transgenic line expressing SR1-YFP using anti-GFP antibody.

Suppl. Fig. S1

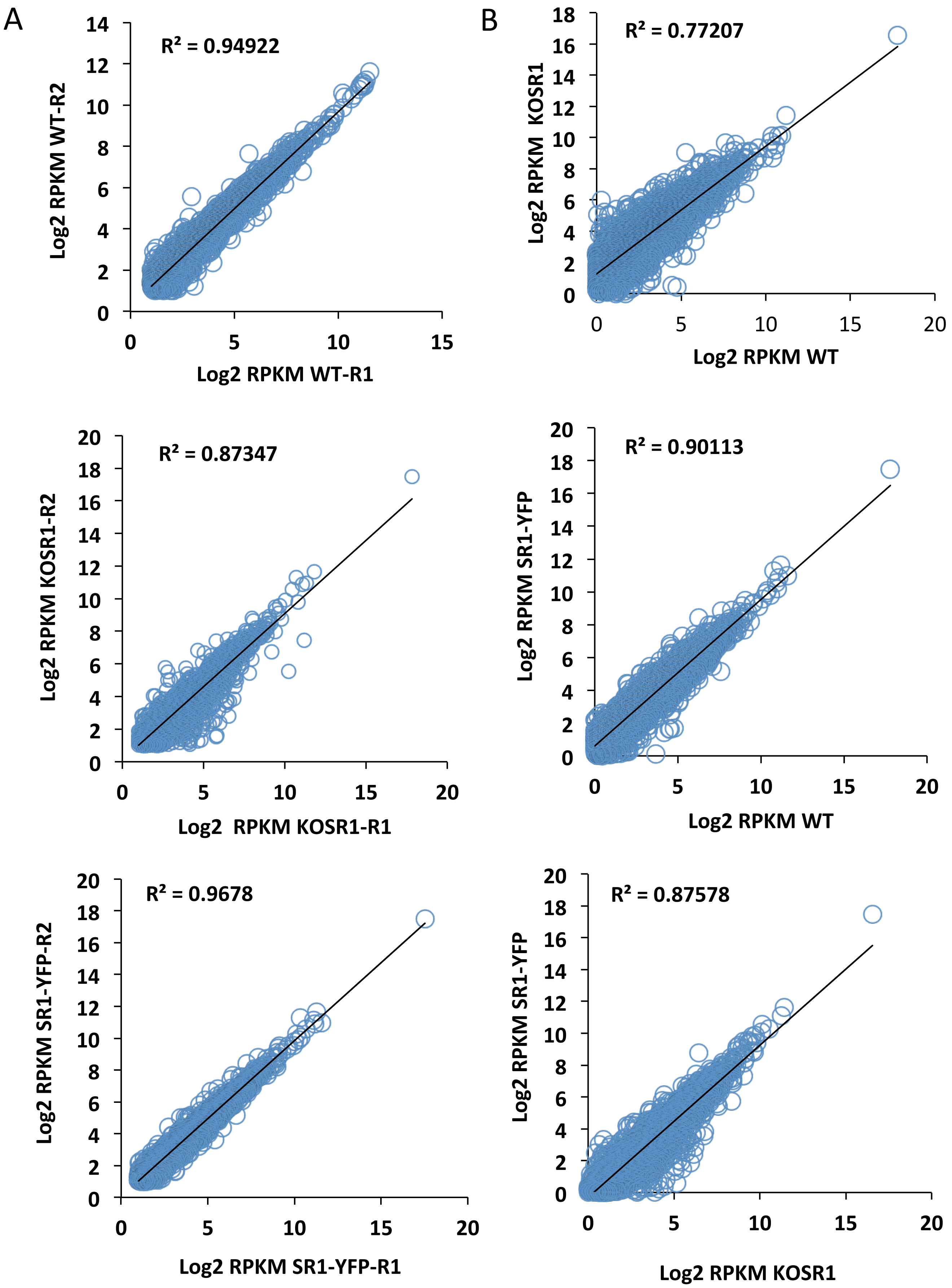
A
R² = 0.94922
Log2 RPKM WT-R2
Log2 RPKM WT-R1
B
R² = 0.77207
Log2 RPKM KOSR1
Log2 RPKM WT
R² = 0.87347
Log2 RPKM KOSR1-R2
Log2 RPKM KOSR1-R1
R² = 0.90113
Log2 RPKM SR1-YFP
Log2 RPKM WT
R² = 0.9678
Log2 RPKM SR1-YFP-R2
Log2 RPKM SR1-YFP-R1
R² = 0.87578
Log2 RPKM SR1-YFP
Log2 RPKM KOSR1

Suppl. Fig. S2

Supplementary Figure S2. Scatter plot of RPKM values between replicates or genotypes. The Log2 transformed values of replicates of wild type, *sr1-1* and SR1-YFP (panel A) and between the genotypes of wild type, *sr1-1* and *SR1-YFP* (panel B) are plotted.

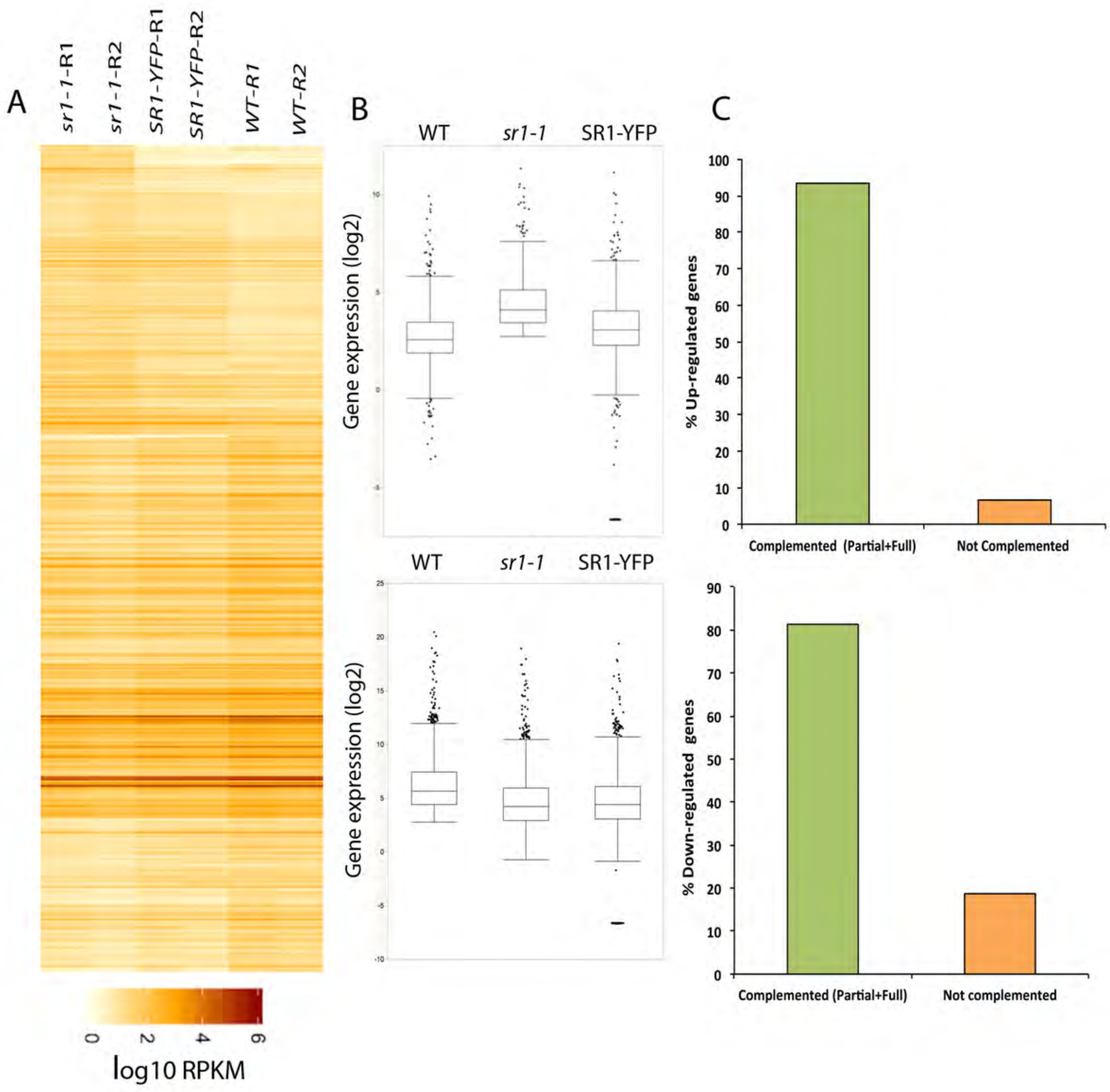

Suppl. Fig. S3

Supplementary Figure S3. **Differentially expressed genes in WT, *sr1-1* and SR1-YFP plants.**
A) Heatmap of DE genes in two biological replicates of WT, *sr1-1* and SR1-YFP plants. RPKM values were used to generate the heatmap with CummeRbund1. **B)** Box-and-whisker plots showing expression of up- (top panel) and down-regulated (bottom panel) DE genes in different genotypes. **C)** Percentage of up- (top panel) and down-regulated (bottom panel) DE genes that are either fully or partially complemented in SR1-YFP line.

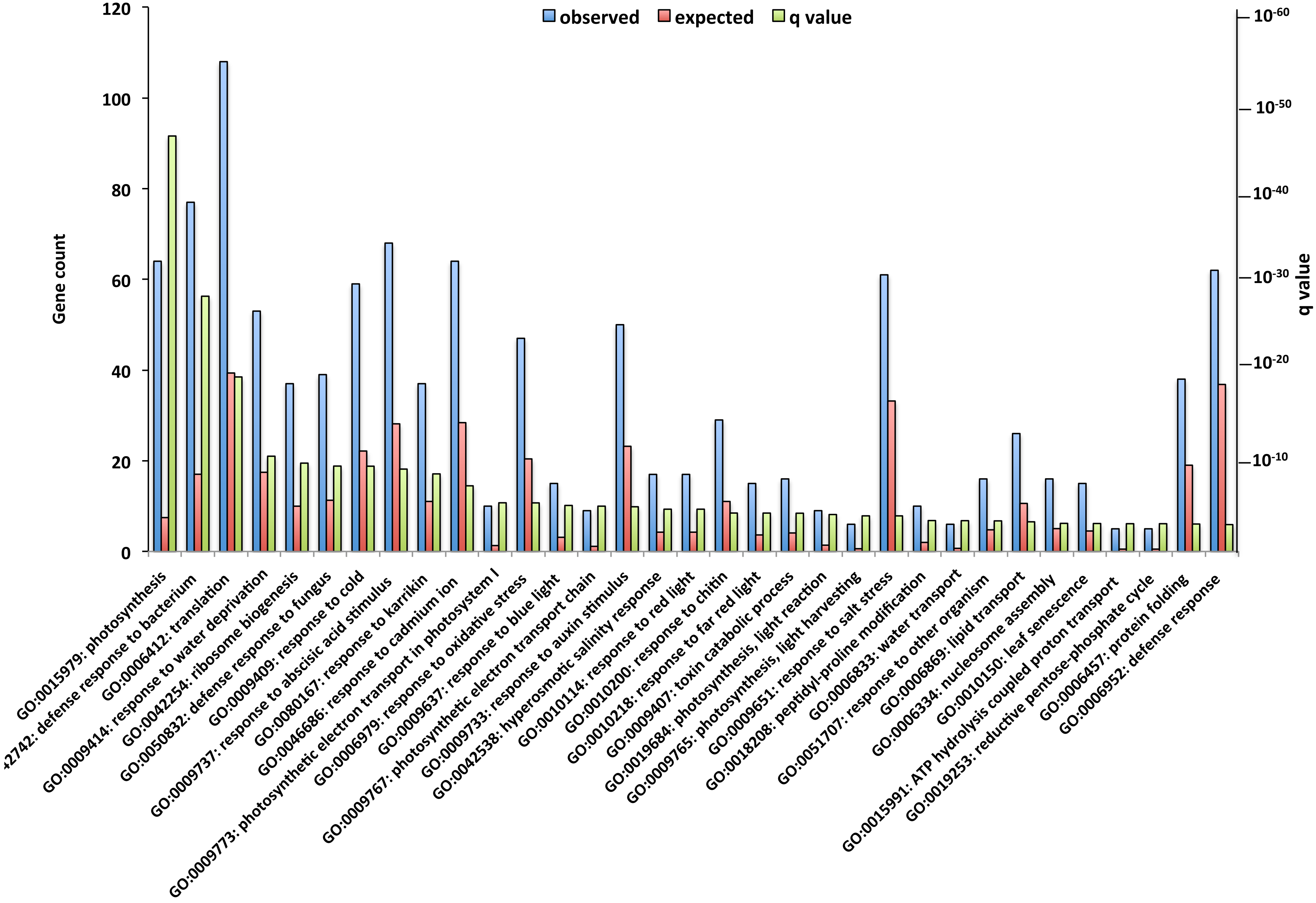

Suppl. Fig. S4A

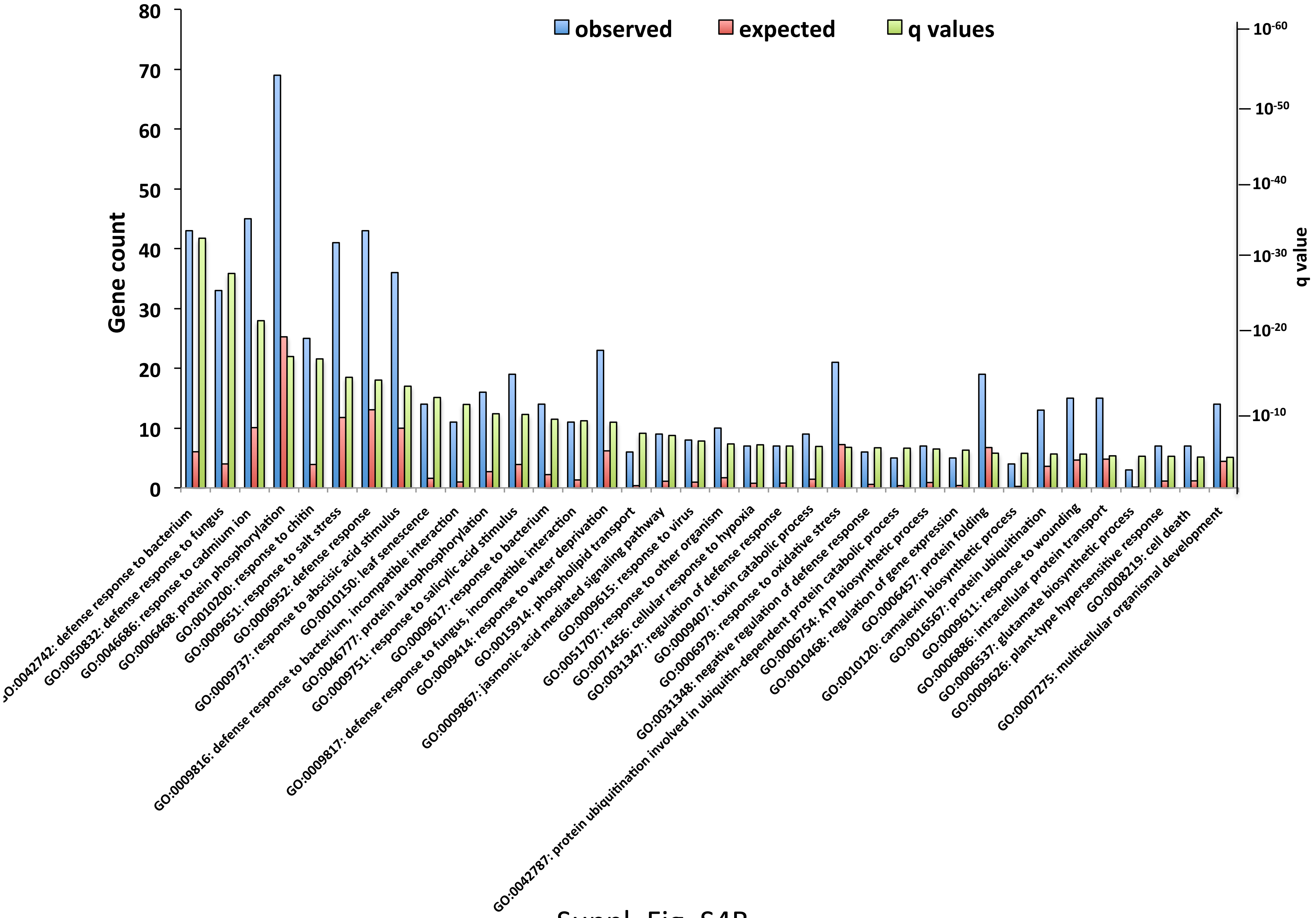

Suppl. Fig. S4B

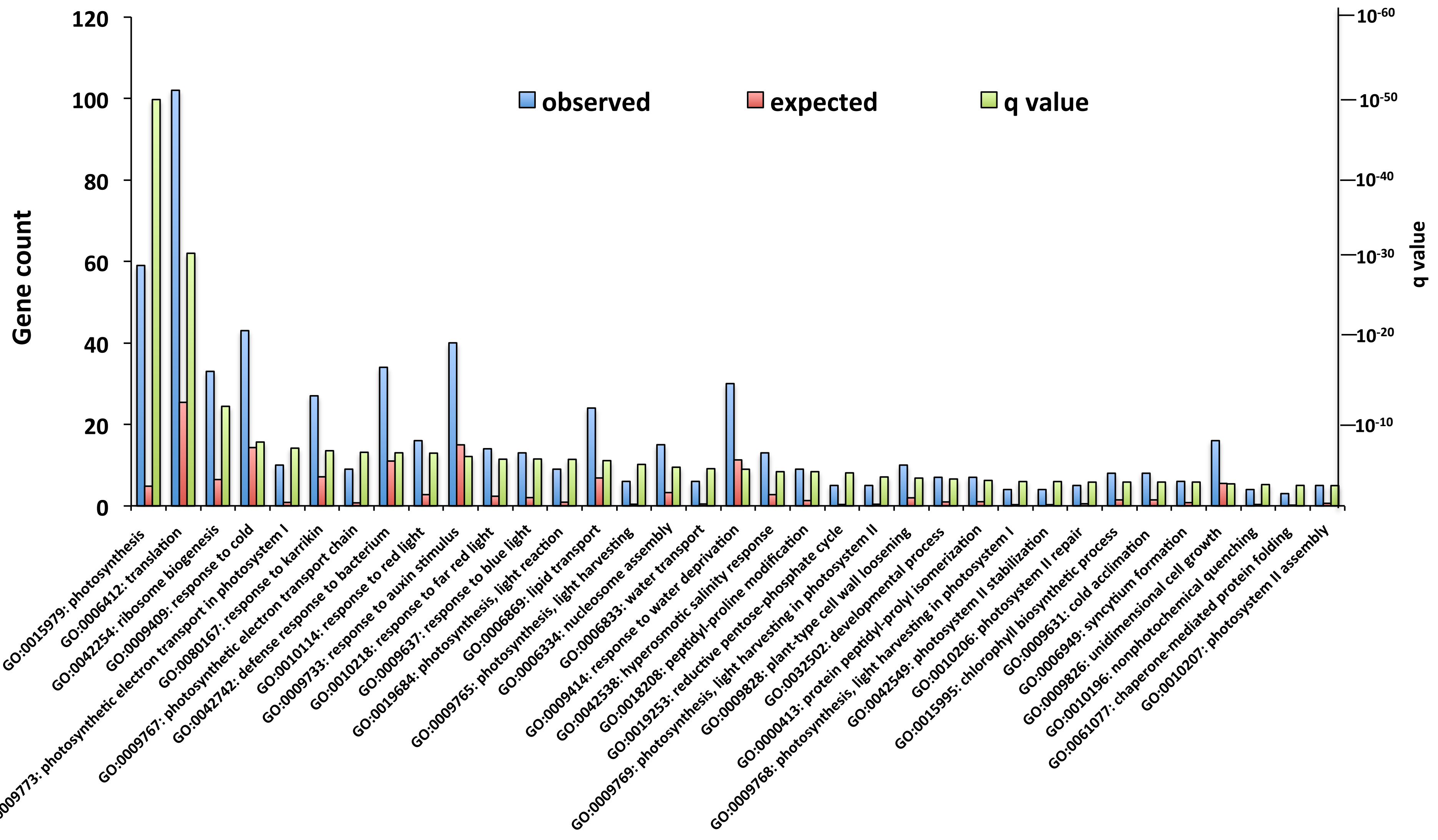

Suppl. Fig. S4C

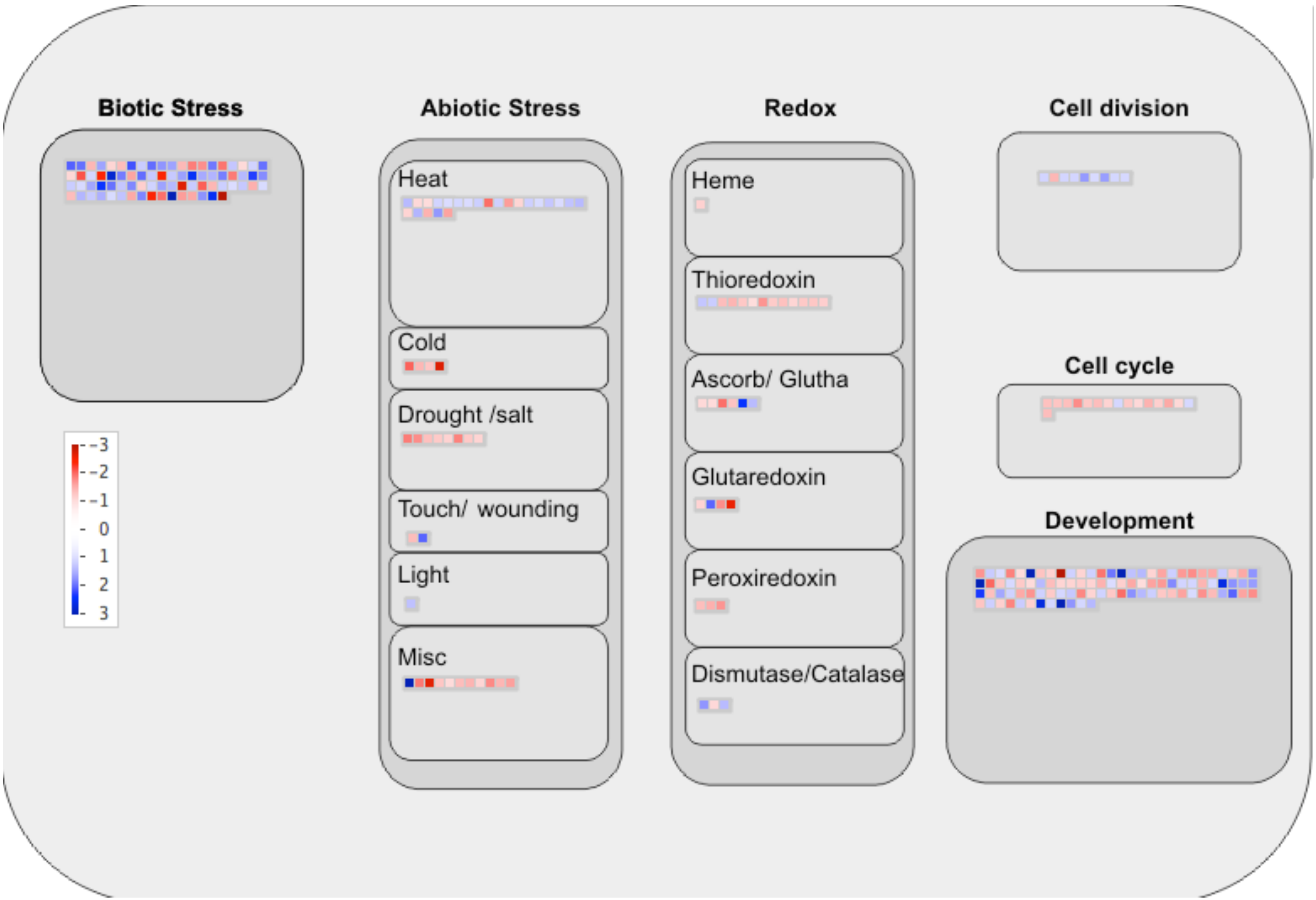

Suppl. Fig. S4D

Supplementary Figure S4. GO term enrichment analysis. GO term enrichment analysis for biological processes of **A)** all DE, **B)** up-regulated and **C)** down-regulated genes. For each GO term, the expected and observed gene numbers along with the statistical significance (q-value) for the enrichment is presented. Observed: Number of DE genes with a GO term for biological processes. Expected: Number of genes expected for each GO term in the whole genome. "Response to salt stress" GO term is indicated with an arrow. **D)** DE genes that are involved in "cellular response" categories were visualized using the MapMan software2. Red indicates decrease whereas blue indicates increase in expression of DE genes in the mutant.

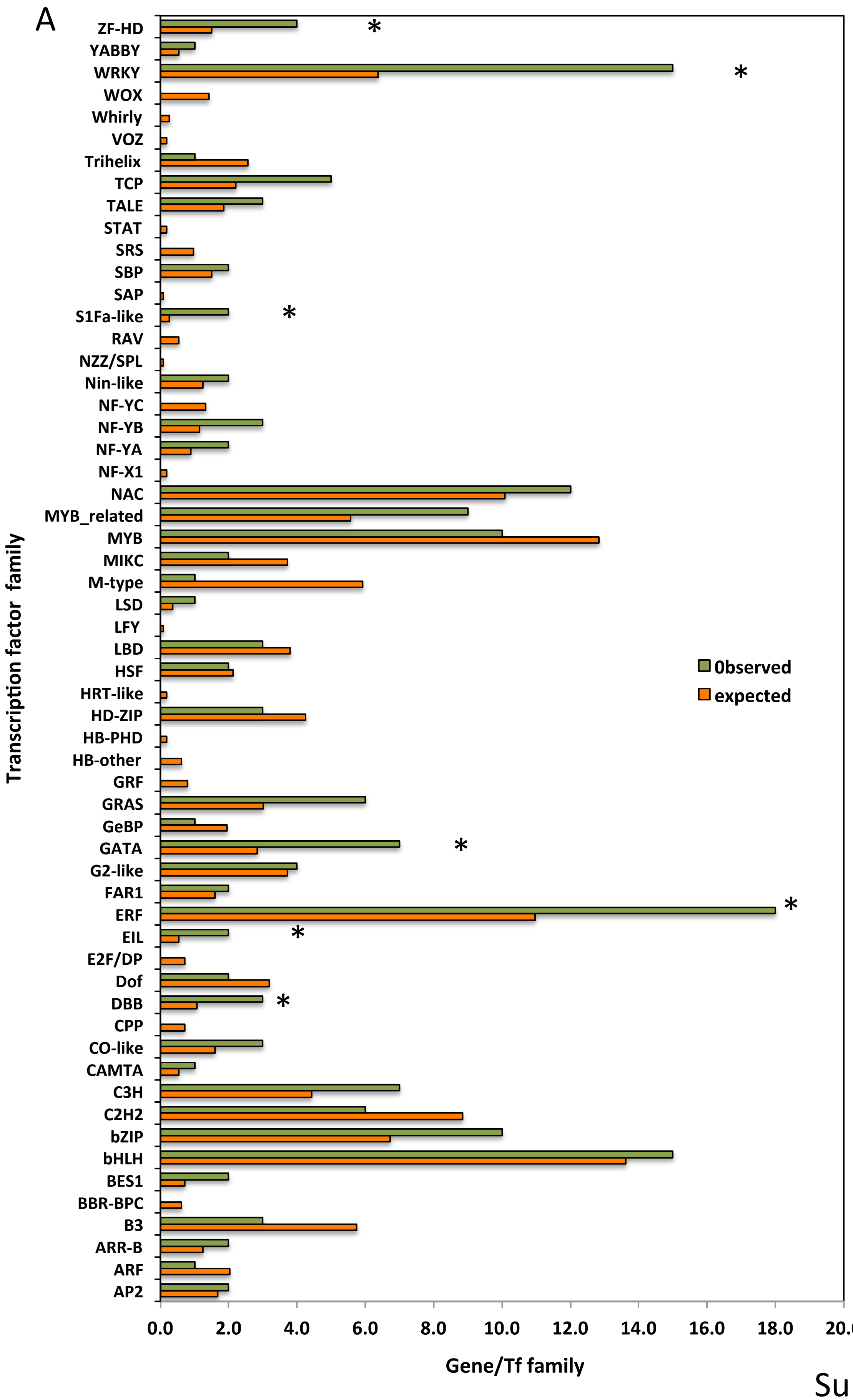

Suppl. Fig. S5A

B

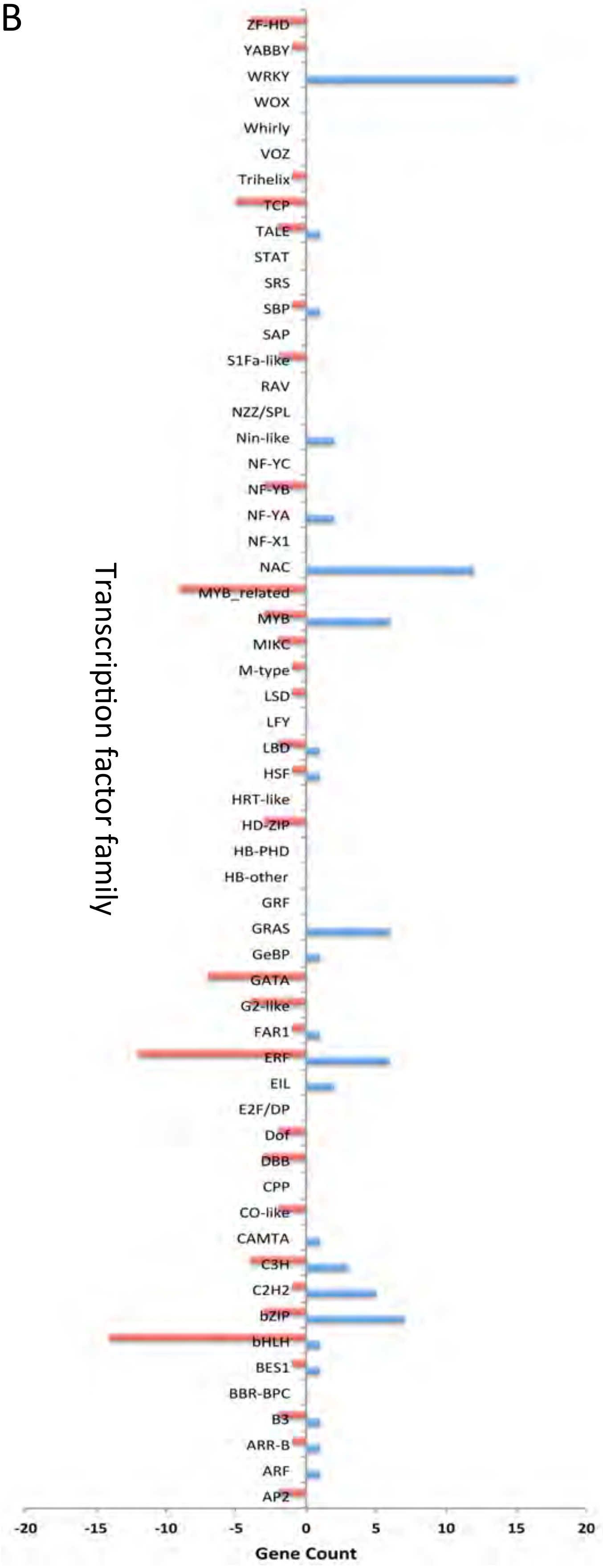

Suppl. Fig. S5B

Supplementary Figure S5. TF gene families in DE genes A) Enrichment of TF families in all DE genes. DE genes are enriched ($P < 0.05$) for specific TF families, which are indicated with an asterisk. Observed: Number of genes associated with particular TF family in DE genes. Expected: Number of genes expected in each individual TF family in the genome. **B)** Up- and down-regulated DE genes in each TF family.

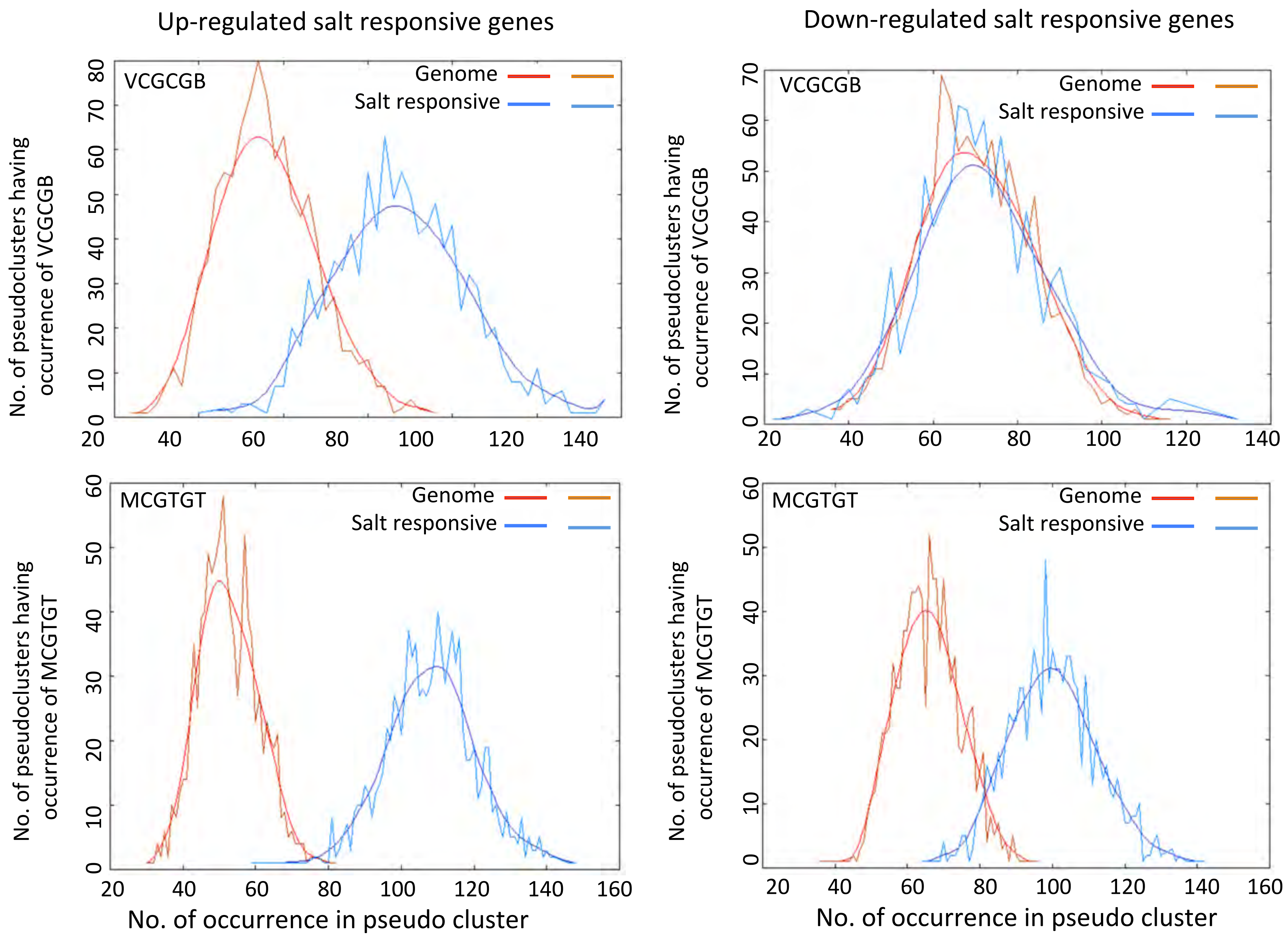

Suppl. Fig. S6A

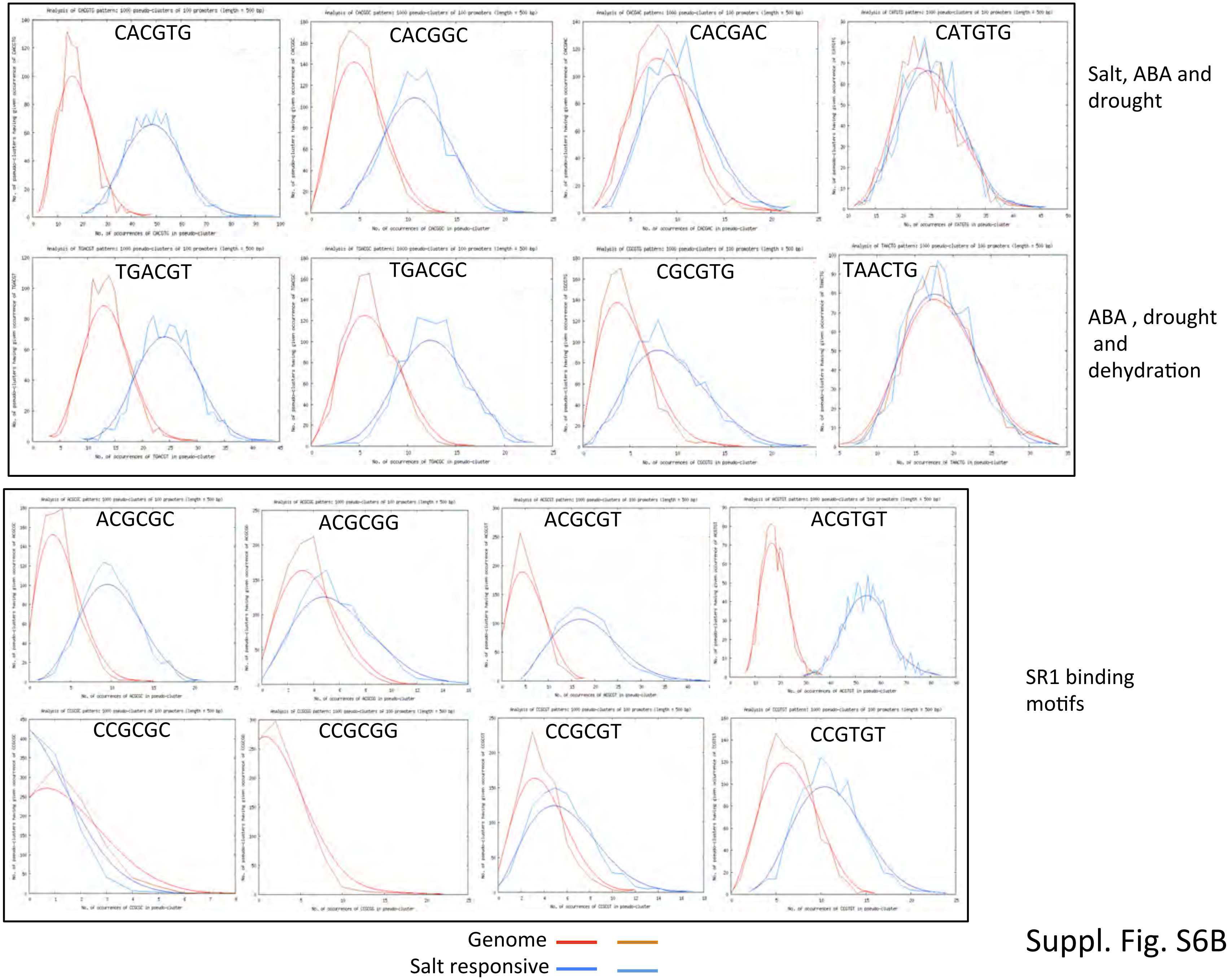

Suppl. Fig. S6B

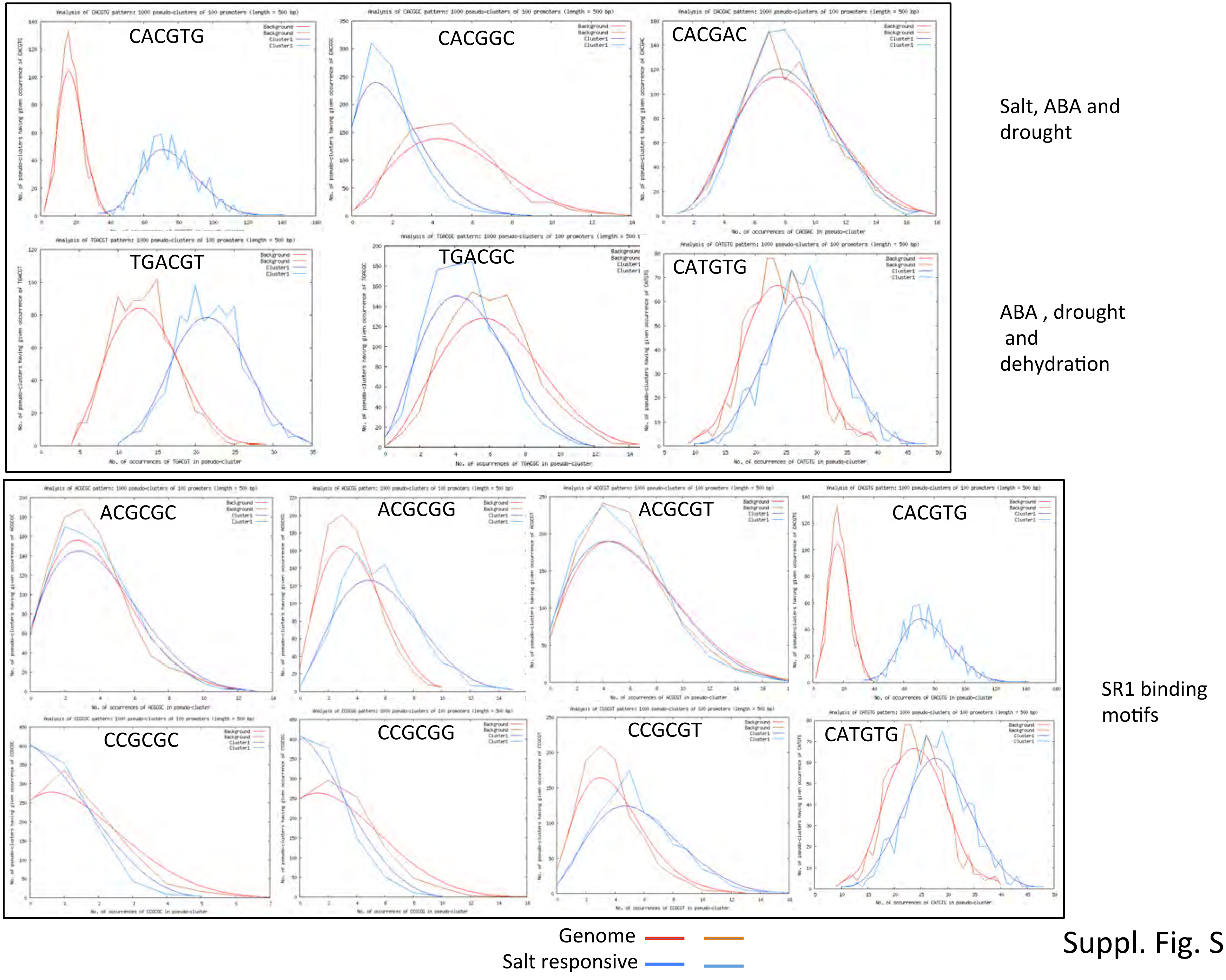

Suppl. Fig. S6C

Supplementary Figure S6. Promoter analysis of differentially regulated salt-responsive genes.

A) POBO analysis indicating the occurrence of the *RSRE* element (*VCGCGB*) and *MCGTGT* in the upstream (-1000 bp from TSS) of salt-responsive DE genes. Data pertaining to 114 up-regulated and 144 down-regulated genes were plotted. A significant (two tailed $P < 0.0001$) enrichment of *VCGCGB* motif was found only in the promoter regions of up-regulated genes

B) POBO analysis calculating the occurrence of salt-specific *cis*-elements in the upstream (500 bp of TSS) of salt-responsive 114 up-regulated genes (Top panel). Occurrence of SR1 binding motifs was also plotted (Bottom panel). A significant (two tailed $P < 0.0001$) enrichment of *CACGTG (G-Box)* and *CACGGC (N-box)* motifs was observed in the promoter regions of up-regulated genes.

C) POBO analysis calculating the occurrence of salt specific *cis*-elements in the upstream (500 bp of TSS) of salt-responsive down-regulated genes (Top panel). Occurrence *SR1* recognition motifs was also plotted (Bottom panel). Data pertaining to 144 down-regulated genes were plotted. A significant (two tailed $P < 0.0001$) enrichment of only *CACGTG (G-Box)* motif was found in the promoter regions of down-regulated genes.

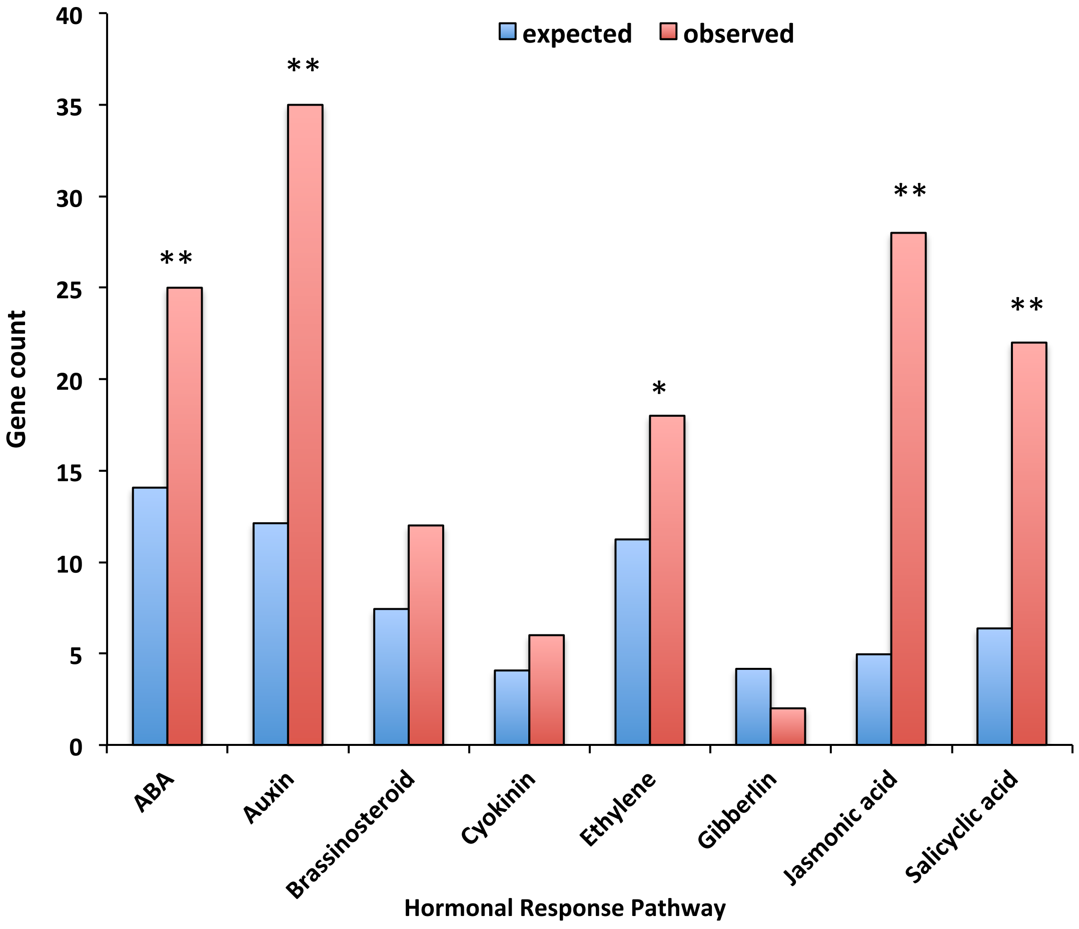

Suppl. Fig. S7

Supplementary Figure S7. Enrichment of phytohormone-response pathways in DE genes. DE genes were enriched for various phytohormone-response pathways. Observed: Number of genes associated with each individual hormonal pathway. Expected: Number of genes expected to associate with each individual hormonal pathway in the whole genome. Asterisks above each bar represent significance level (** for $P < 0.0001$ and * $P < 0.05$).

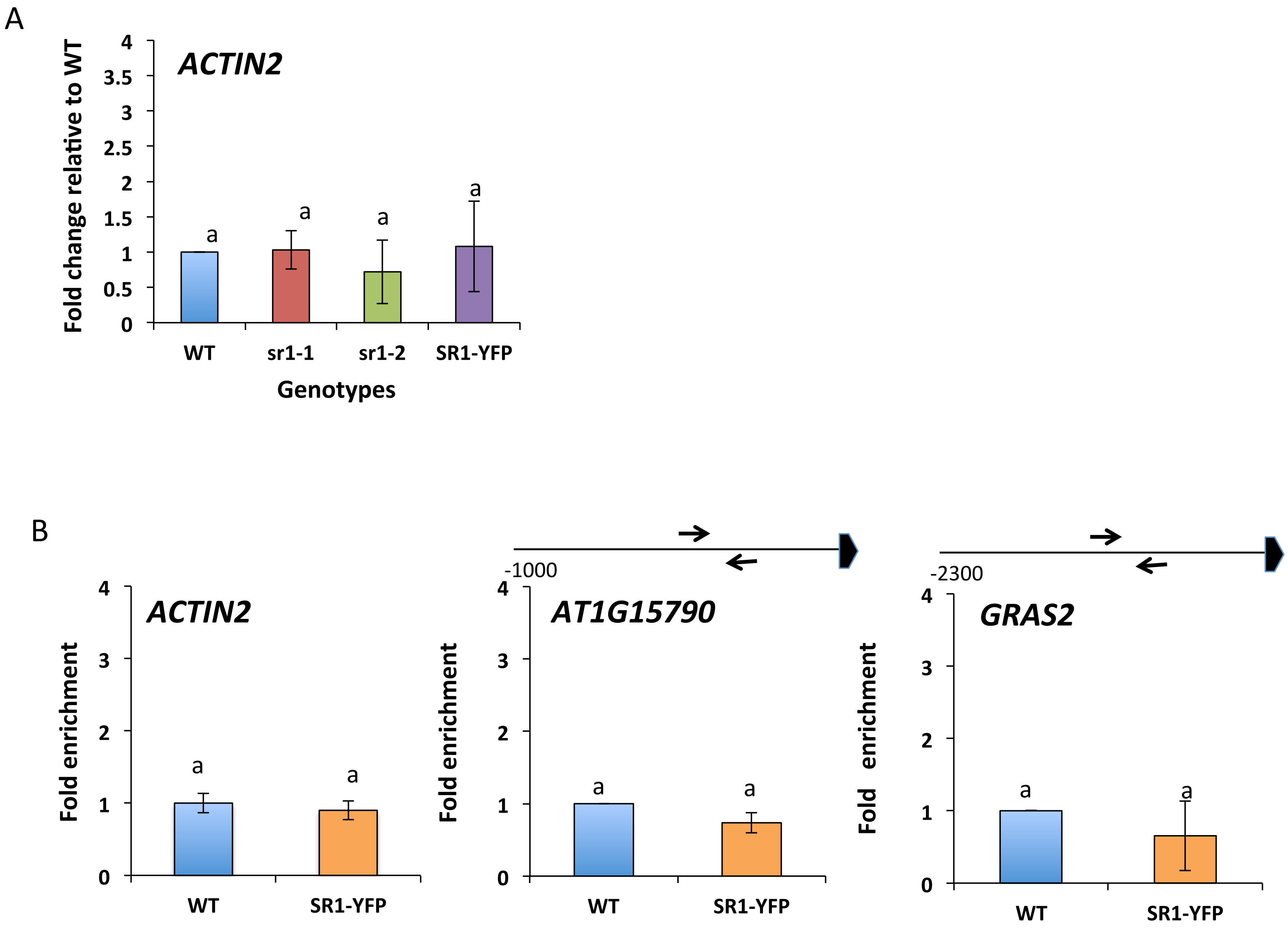

A
ACTIN2
Fold change relative to WT
4
3.5
3
2.5
2
1.5
1
0.5
0
a
a
a
a
WT
sr1-1
sr1-2
SR1-YFP
Genotypes
B
ACTIN2
Fold enrichment
4
3
2
1
0
a
a
WT
SR1-YFP
-1000
AT1G15790
Fold enrichment
4
3
2
1
0
a
a
WT
SR1-YFP
-2300
GRAS2
Fold enrichment
4
3
2
1
0
a
a
WT
SR1-YFP

Suppl. Fig. S8

Supplementary Figure S8. A) Expression of *ACTIN2* in all genotypes**. B)** ChIP-PCR of *ACTIN2* and two other genes that do not contain SR1 binding motifs in their promoters. Chromatin from 15-day-old seedlings from WT and SR1-YFP was immunoprecipitated with anti-GFP antibody and used in PCR. The results obtained from four independent ChIP experiments were used to calculate fold enrichment. In case of *ACTIN2* that data were normalized to DNA input levels. In case of *At1g15790* and *GRAS2* promoters, the data were normalized with DNA input and *ACTIN2*. The values of WT were considered as 1. Student t-test was performed and significant differences ($P<0.05$) among samples are labeled with different letters. Schematic diagram over the panel shows the location of primers (indicated by arrows) used in ChIP-PCR. Bold arrowhead indicates TSS.

1 Trapnell, C. *et al.* Differential gene and transcript expression analysis of RNA-seq experiments with TopHat and Cufflinks. *Nature protocols* **7**, 562-578 (2012).

2 Usadel, B. *et al.* A guide to using MapMan to visualize and compare Omics data in plants: a case study in the crop species, Maize. *Plant Cell and Environment* **32**, 1211-1229, doi:10.1111/j.1365-3040.2009.01978.x (2009).
